# Adverse childhood and adulthood experiences and risk of new-onset cardiovascular disease with consideration of social support: a prospective cohort study

**DOI:** 10.1186/s12916-023-03015-1

**Published:** 2023-08-08

**Authors:** Wanxin Wang, Yifeng Liu, Yuwei Yang, Weiqing Jiang, Yanyan Ni, Xue Han, Ciyong Lu, Lan Guo

**Affiliations:** 1 Department of Medical Statistics and Epidemiology, School of Public Health, Sun Yat-Sen University, Guangzhou, 510080 People’s Republic of China; 2 Department of Psychiatry, Shenzhen Nanshan Center for Chronic Disease Control, Shenzhen, People’s Republic of China; 3 Guangdong Provincial Center for Disease Control and Prevention, Guangzhou, People’s Republic of China; 4 School of Public Health, The University of Hong Kong, Hong Kong SAR, People’s Republic of China

**Keywords:** Adverse childhood experience, Adverse adulthood experience, New-onset cardiovascular disease, Cohort study

## Abstract

**Background:**

The relationship between adverse childhood experiences (ACEs) and adverse adulthood experiences (AAEs) and their association with incident cardiovascular disease (CVD) have not been extensively studied. Considering social support, we evaluated the complex relations of ACEs and AAEs with incident CVD.

**Methods:**

This prospective cohort study used data from the 2014 life course survey and the 2015 and 2018 surveys of the China Health and Retirement Longitudinal Study, a national survey of Chinese adults aged ≥ 45 years from 28 provinces across China. The study population included 5836 individuals (mean [SD] age, 59.59 [8.22] years, 49.7% were males). Information on ACEs, AAEs, young adulthood social support, health behavior factors, health status factors, and demographics was measured. Cox regression models, the difference method to estimate the mediation proportion, and the additive and multiplicative interactions were performed. Subgroup and sensitivity analyses were also conducted.

**Results:**

During follow-up, 789 incident cases of CVD occurred. The fully adjusted model, including demographics, health behaviors, health status factors (e.g., depressive symptoms), and social support as control variables, demonstrated that the overall number of ACEs (Hazard ratio [HR]: 1.11, 95% CI: 1.08 to 1.14) and AAEs (HR: 1.19, 95% CI: 1.16 to 1.22) were associated with an increased risk of incident CVD. A dose–response relationship existed between the number of ACEs or AAEs and incident CVD risk. The overall AAEs were found to mediate 17.7% (95% CI: 8.2 to 34.2%) of the association between ACEs and incident CVD. Moreover, a significant additive interaction between ACEs and AAEs was detected (RERI [95% CI]: 0.32 [0.09 to 0.56]). Compared with adults without exposure to both ACE and AAE, those with exposure to both at least one ACE and one AAE indicator had the highest risk of incident CVD (HR: 1.96, 95% CI: 1.72 to 2.23).

**Conclusions:**

Exposure to ACEs or AAEs was independently associated with an increased risk of incident CVD among Chinese middle-aged and older adults in a dose–response manner, and the overall AAEs partially mediated the association between ACEs and incident CVD. Preventive measures aimed at addressing either ACEs or AAEs alone may not significantly reduce the risk of CVD later in life. The necessity of a comprehensive life-course health strategy targeting the prevention of adversity merits increased attention.

**Supplementary Information:**

The online version contains supplementary material available at 10.1186/s12916-023-03015-1.

## Background

Cardiovascular diseases (CVDs) are the leading global cause of mortality and a significant contributor to disability [1]. The World Health Organization defines CVD as a group of disorders of the heart and blood vessels, including stroke, coronary heart disease, and other related conditions [2]. Recent studies have highlighted the association between adverse childhood experiences (ACEs) and a series of cardiovascular risk factors [3, 4] and events later in life [5–7]. ACEs refer to a broad range of potentially stressful experiences during childhood and adolescence [8]. The American Heart Association (AHA) has issued scientific and policy statements recognizing the impact of ACEs on cardiometabolic health throughout the life course [9, 10]. The AHA statement proposes potential pathways that link ACEs and CVD by affecting health behaviors, such as smoking and physical inactivity, pathophysiological factors like the dysregulation of the nervous, immune system, and neuroendocrine systems, and psychological factors [10]. These pathways have been supported by a recent review [5] and several studies [11, 12].

In addition, exposure to ACEs may heighten the risk of encountering adversities and perceived burdensomeness in adulthood [13, 14]; and adverse adulthood experiences (AAEs) may act as triggers in the association between ACEs and adult health [15]. However, critical knowledge gaps remain. First, few studies have concurrently investigated the impacts of ACEs and AAEs on subsequent CVDs. Research on the interaction and joint associations of ACEs and AAEs with cardiovascular health is limited, and results are inconsistent. A recent cross-sectional study revealed that childhood adversity and adulthood stressful life events are significantly and independently linked to cardiovascular health in German adults, without interactions between childhood adversity and adulthood stressful life events [16]. In contrast, a previous longitudinal study using a single variable (i.e., adulthood neighborhood disadvantage) to represent individual-level AAEs found the combined effects of childhood psychosocial adversity and neighborhood disadvantage on incident CVD among Finnish adults [7]. Second, although AAEs may be mediators between ACEs and CVDs, few studies have constructed pathway models linking ACEs, AAEs, and subsequent CVDs, and the extent to which the overall AAEs mediate the association between ACEs and incident CVD remains to be explored. Third, it is unclear whether the findings are consistent across subpopulations of different ages and sex.

According to the social support models proposed by Cohen and Wills, good social support can directly or indirectly mitigate the negative impacts of stressful events through both its structural aspects, such as the existence of relationships, and its functional aspects, which involve perceived supports that address the needs arising from stressful events [17]. Despite this knowledge, the role of social support in mitigating the adverse effects of ACEs or AAEs on cardiometabolic health has not received sufficient attention [18, 19]. Therefore, it is hypothesized that social support may serve as a covariate or modifier in the associations of ACEs and AAEs with incident CVD. Using data from the China Health and Retirement Longitudinal Study (CHARLS) and taking into account the influence of social support, this study has three main objectives: firstly, to investigate the associations of ACEs and AAEs with incident CVD; secondly, to estimate the extent to which the overall AAEs mediate the association between ACEs and incident CVD; and finally, to explore potential interactions or joint relations of ACEs and AAEs on incident CVD.

## Methods

### Study population

**Fig. 1:**
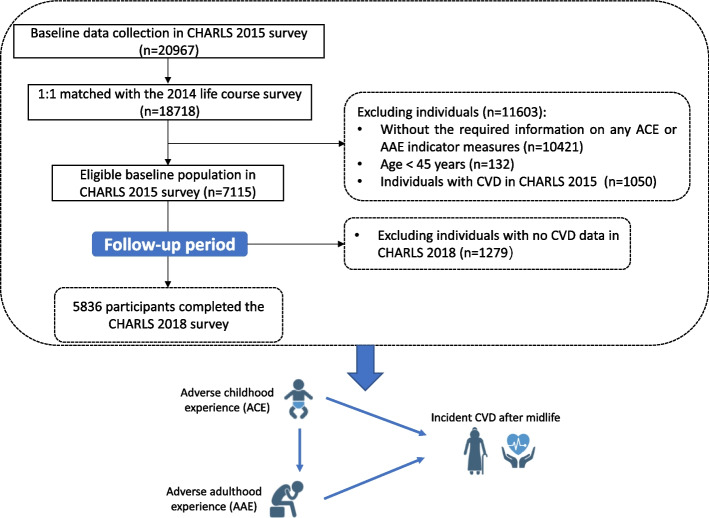
Flowchart depicting the study design and derivation of the study population

This cohort study was a secondary analysis of the data from the CHARLS, an ongoing nationally representative cohort study among Chinese adults aged ≥ 45 years from 450 villages or resident communities within 28 provinces across China [20]. The study has conducted four main surveys, with the first wave being launched in 2011, and follow-up surveys in 2013, 2015, and 2018. Information on ACEs, AAEs, and young adulthood social support was additionally collected during the 2014 life course survey among all living respondents in the 2011 and 2013 waves surveys. The details of the CHARLS survey have been previously reported [20, 21]. The current study utilized data from the 2014 life course survey and the 2015 and 2018 main surveys of CHARLS. As described in the Additional file 1: Appendix. S1 and Fig. 1, 7115 adults in the 2015 main survey (baseline) have information from the 2014 life course survey. We excluded those without the required information on any ACE or AAE indicator measures (*N* = 10,421), aged below 45 years or without age information (*N* = 132), with CVD in the CHARLS 2015 survey (*N* = 1050), and with no CVD data in the CHARLS 2018 survey (*N* = 1279); our final cohort comprised 5836 individuals.

### Measures

#### Adverse childhood and adulthood experiences

In accordance with previous literature about ACE that established their significant implications for health and social outcomes [5, 8, 22, 23], this study extracted ten widely used ACE indicators. Briefly, we defined three domains of ACEs, including the household dysfunction domain (household substance use, household mental illness, domestic violence, criminal behavior in the household, parental separation or divorce, and parental death), neglect and abuse domains (physical neglect, emotional neglect, and physical abuse), and additional ACE domain (exposure to natural disasters). All ACE indicators are defined in Additional file 2: Tab. S1, and responses to each indicator were dichotomized. The ten indicators were summed to generate a cumulative ACE score for each individual, resulting in a cumulative score ranging from 0 to 10. Based on the cumulative numbers of ACE, participants were categorized into five groups: 0, 1, 2, 3, and 4 or more ACEs [5, 23]. Based on previous literature [24], this study extracted five AAE indicators from the CHARLS dataset, including experiencing the death of the child, lifetime discrimination, ever being confined to bed, ever being hospitalized for a month or longer, and ever leaving a job due to health conditions. The details of questions and responses for each factor are outlined in Additional file 2: Tab. S1. Responses to each indicator were dichotomized and summed to generate a cumulative AAE score for each individual, ranging from 0 to 5. Participants were further classified into five groups based on the cumulative numbers of AAE: 0, 1, 2, 3, and 4 or more AAEs. The assessment of ACE or AAE has been extensively employed in previous studies [5, 8, 10, 12, 15, 24–28].

#### Ascertainment of incident CVD events

Following previous research utilizing the CHARLS dataset [29, 30], the study outcome was incident CVD events during the follow-up period, and the following standardized questions assessed incident CVD events: “Have you been told by a doctor that you have been diagnosed with a heart attack, angina, coronary heart disease, heart failure, or other heart problems?” or “Have you been told by a doctor that you have been diagnosed with a stroke?”. Participants reporting heart disease or stroke during the follow-up period were defined as having incident CVD. The follow-up time was measured as the time elapsed from the date of the last interview to either the date of CVD diagnosis or the date of the latest interview (March 2019) in which the individual participated.

#### Other covariates

Demographic and health-related data were collected through face-to-face interviews. Demographic information included age, sex (male or female), residential area (rural or urban), marital status (married or other marital status, including never married, separated, divorced, and widowed), and educational level (no formal education, primary school or below, middle or high school, or college or above). Health behavior factors included smoking and alcohol consumption (never, former, or current) and physical activity. Health status factors consisted of body mass index (BMI), depressive symptoms, self-reported physician-diagnosed health conditions (hypertension, diabetes, dyslipidemia, and chronic kidney disease), and the use of medications or therapies for hypertension, diabetes, and dyslipidemia. Further details on the definitions of these covariates can be found in Additional file 1: Appendix. S1 [31–33].

Young adulthood social support, encompassing economic, noneconomic, and emotional support, was also assessed in the CHARLS dataset through the following questions [34], “when you were a young adult, was there anyone who provided you with financial support for your work?”, “when you were a young adult, was there anyone who provided you with positive nonfinancial support for work?”, and “when you were a young adult, was there anyone who provided you with positive support or mentoring for your interpersonal relationship?”. Responses were dichotomized, and a total score ranging from 0 to 3 was calculated, with higher scores indicating greater social support [34].

A sub-cohort of 4113 individuals underwent assessments of metabolic biomarkers. These included fasting plasma glucose, glycosylated hemoglobin (HbA1c), total cholesterol, triglycerides, high-density lipoprotein cholesterol, low-density lipoprotein cholesterol, high-sensitivity C-reactive protein (hs-CRP), and serum creatinine. The estimated glomerular filtration rate (eGFR) was calculated using the 2009 creatinine equation from the Chronic Kidney Disease Epidemiology Collaboration [35].

#### Statistical analysis

Descriptive statistics are presented as means with standard deviations (SDs) or median with interquartile range (IQR) for continuous variables and frequency with percentage for categorical variables. First, the baseline characteristics were summarized for different levels of ACEs and AAEs, and differences among groups were tested using appropriate statistical tests such as the chi-square test, analysis of variance, or Kruskal–Wallis test.

Second, in this study, 837 of 5836 data items were missing and were imputed using the multiple imputations of chained equations method with baseline characteristics. Ten imputed data sets were created, and the main analyses were applied to these sets [36]. The incidence rates of CVD per 1000 person-years were calculated, and Cox proportional hazard regression models were used to estimate the hazard ratios (HRs) and 95% confidence intervals (CIs) of outcomes associated with ACE and AAE levels. Four models were estimated based on previous literature [29, 37], where model 1 was adjusted for age and sex; model 2 was adjusted for age, sex, residence, marital status, educational level, and health behaviors (physical activity, smoking status, and alcohol consumption); model 3 was adjusted for the variables in model 2 plus health status variables (BMI; history of diabetes, hypertension, dyslipidemia, and chronic kidney disease; use of diabetes medications, hypertension medications, and lipid-lowering therapy; and depressive symptoms); model 4 was adjusted for the variables in model 3 and additionally added young adulthood social support.

Third, to explore the role of AAEs in the ACEs and incident CVD relationship, we employed the difference method to estimate the proportion of mediation by the overall AAEs for the association between ACEs and incident CVD by comparing the estimates from models without and with the hypothesized mediator [38]. Additionally, we calculated the C statistics of the two models to compare the predictions with and without adjusting for the overall AAEs [39].

Fourth, we examined the joint associations of ACEs and AAEs by categorizing ACEs and AAEs levels into two categories: those who did not report any ACE or AAE (0) and those who reported at least one ACE or AAE (≥ 1 indicator). Participants were then divided into four groups based on their combined ACEs and AAEs status. To assess the additive and multiplicative interactions between ACEs and AAEs, we included a product interaction term (ACEs × AAEs) in model 4, and the HR (95% CI) of the product term was used to measure interaction on the multiplicative scale. The relative excess risk due to interaction (RERI) with 95% CI was also calculated as a measure of interaction on the additive scale, using the regression coefficients and covariance matrix [40].

Young adulthood social support, sex, and age were also tested as effect modifiers in models 1 through 3, with a product interaction term (ACEs/ AAEs × young adulthood social support/sex/age) fitted. Moreover, to explore potential variations in different subgroups, we conducted subgroup analyses by sex (males and females) and age groups (< 60 years and ≥ 60 years, defined as elders by the World Health Organization [41]).

Three sensitivity analyses were also performed to test the robustness of the findings: (1) we further adjusted for metabolic biomarkers in the subgroup of 4113 participants who underwent metabolic examinations; (2) we used each ACE or AAE indicator separately instead of the overall accumulation of ACEs or AAEs indicators in the models. This allowed us to determine if the results remained consistent with those of the main analyses and provided additional insights into which specific ACEs or AAEs might have significant impacts on incident CVD; (3) we repeated all analyses using the complete dataset (4996 participants).

All analyses were conducted using SAS (version 9.3, SAS Institute, Cary, NC) and Stata (version 17.0, StataCorp, College Station, TX, USA). Two-sided *P* < 0.05 was considered statistically significant.

## Results

### Characteristics of the study population

Table 1 depicts the baseline characteristics of the study population stratified by the number of ACEs, while Additional file 2: Tab. S2 presents the baseline characteristics stratified by the number of AAEs. The study included 5836 participants, with a mean (SD) age of 59.59 (8.22) years, and 49.7% were males. Of the total population, 80.5% had been exposed to at least one ACE indicator, 36.2% had been exposed to at least one AAE indicator, and 30.9% had been exposed to both ACEs and AAEs, shown in Additional file 3: Fig. S1. Participants exposed to one or more ACEs or AAEs indicators were more likely to be older, living in rural areas, unmarried, less educated, current smokers/drinkers, and have higher depressive symptoms scores than those without exposure. We also observed an increasing trend in the prevalence rates of hypertension and chronic kidney disease as the number of ACEs indicators increased.

**Table 1:** Baseline characteristics of the study population by the number of adverse childhood experiences (ACEs)

Characteristics	Total	Number of ACEs indicators (*N* = 5836)					
		**0 (*n* = 1137)**	**1 (*n* = 2095)**	**2 (*n* = 1388)**	**3 (*n* = 821)**	** ≥ 4 (*n* = 395)**	***P* value**^**a**^
Age, mean (SD), years	59.6 (8.2)	56.3 (8.3)	59.8 (7.9)	60.7 (8.0)	60.8 (8.2)	61.4 (7.9)	< 0.001
Sex, *n* (%)							
Male	2901 (49.7)	486 (42.7)	1080 (51.6)	723 (52.1)	414 (50.4)	198 (50.1)	< 0.001
Female	2935 (50.3)	651 (57.3)	1015 (48.4)	665 (47.9)	407 (49.6)	197 (49.9)	
Residential area^b^, *n* (%)							
Rural	4799 (82.2)	879 (77.3)	1677 (80.0)	1187 (85.6)	704 (85.7)	352 (89.1)	< 0.001
Urban	1036 (17.8)	258 (22.7)	418 (20.0)	200 (14.4)	117 (14.3)	43 (10.9)	
Marital status							
Married	4972 (85.2)	995 (87.5)	1808 (86.3)	1166 (84.0)	681 (82.9)	322 (81.5)	0.003
Other marital status	864 (14.8)	142 (12.5)	287 (13.7)	222 (16.0)	140 (17.1)	73 (18.5)	
Educational level^b^, *n* (%)							
No formal education	2288 (39.2)	315 (27.7)	737 (35.2)	599 (43.2)	417 (50.8)	220 (55.7)	< 0.001^e^
Primary school or below	1348 (23.1)	231 (20.3)	504 (24.1)	342 (24.7)	185 (22.5)	86 (21.8)	
Middle or high school	2079 (35.6)	548 (48.2)	804 (38.4)	429 (30.9)	213 (25.9)	85 (21.5)	
College or above	117 (2.0)	43 (3.8)	47 (2.2)	17 (1.2)	6 (0.7)	4 (1.0)	
Smoking status^b^, *n* (%)							
Nonsmoker	3509 (62.2)	770 (69.8)	1229 (60.6)	792 (59.3)	484 (60.9)	234 (61.3)	< 0.001
Former smoker	398 (7.1)	60 (5.4)	142 (7.0)	107 (8.0)	58 (7.3)	31 (8.1)	
Current smoker	1735 (30.8)	273 (24.8)	656 (32.4)	436 (32.7)	253 (31.8)	117 (30.6)	
Alcohol consumption^b^, *n* (%)							
Nondrinker	3399 (58.7)	705 (62.7)	1245 (59.9)	777 (56.2)	452 (55.3)	220 (56.0)	0.011
Former drinker	504 (8.7)	92 (8.2)	176 (8.5)	115 (8.3)	84 (10.3)	37 (9.4)	
Current drinker	1891 (32.6)	328 (29.2)	656 (31.6)	490 (35.5)	281 (34.4)	136 (34.6)	
Physical activity (≥ 3 × a week)^b^, *n* (%)							
Yes	4939 (84.6)	973 (85.6)	1786 (85.3)	1182 (85.2)	677 (82.5)	321 (81.3)	0.088
No	897 (15.4)	164 (14.4)	309 (14.7)	206 (14.8)	144 (17.5)	74 (18.7)	
Health conditions (yes)^b^, *n* (%)							
Diabetes	258 (4.5)	51 (4.6)	87 (4.2)	69 (5.1)	33 (4.1)	18 (4.6)	0.787
Hypertension	1094 (19.0)	184 (16.4)	380 (18.4)	290 (21.1)	161 (19.8)	79 (20.3)	0.038
Dyslipidemia	407 (7.2)	83 (7.5)	149 (7.3)	92 (6.8)	48 (6.0)	35 (9.1)	0.355
Chronic kidney disease	333 (5.8)	40 (3.6)	99 (4.8)	97 (7.0)	58 (7.1)	39 (9.9)	< 0.001
History of medication use (yes)^b^, *n* (%)							
Diabetes medications	181 (3.1)	30 (2.6)	64 (3.1)	45 (3.2)	25 (3.0)	17 (4.3)	0.587
Hypertension medications	812 (13.9)	135 (11.9)	301 (14.4)	208 (15.0)	110 (13.4)	58 (14.7)	0.198
Lipid-lowering therapy	213 (3.6)	38 (3.6)	79 (4.0)	49 (3.7)	29 (3.7)	18 (4.9)	0.836
BMI^b^, kg/m^2^, mean (SD)	23.6 (4.2)	23.9 (3.9)	23.8 (4.3)	23.4 (4.2)	23.3 (4.0)	22.9 (4.1)	< 0.001
Blood pressure^b^, mm Hg, mean (SD)							
Systolic pressure	128.1 (21.4)	125.4 (18.6)	128.2 (21.3)	129.1 (21.2)	128.3 (21.3)	130.5 (25.7)	0.002
Diastolic pressure	75.4 (11.9)	75.2 (11.6)	75.5 (11.7)	75.4 (12.1)	74.8 (12.1)	76.0 (12.2)	0.560
Depressive symptoms score^c^, mean (SD)	7.8 (6.1)	6.5 (5.6)	7.1 (5.7)	8.2 (6.1)	9.4 (6.7)	10.61 (6.39)	< 0.001
Adulthood social support, mean (SD)	0.4 (0.7)	0.4 (0.7)	0.3 (0.7)	0.4 (0.7)	0.4 (0.7)	0.4 (0.7)	0.021
Metabolic biomarkers^d^							
Fasting plasma glucose, mg/dL mean (SD)	109.1 (34.1)	108.1 (32.2)	109.5 (36.9)	110.3 (33.3)	107.5 (27.7)	109.3 (39.2)	0.474
HbA1c (%), mean (SD)	14.5 (2.3)	14.2 (2.1)	14.5 (2.3)	14.5 (2.2)	14.5 (2.5)	14.7 (2.6)	0.301
Total Cholesterol, mg/dL, mean (SD),	192.6 (37.3)	191.4 (36.4)	193.0 (37.6)	193.1 (37.2)	191.1 (36.7)	195.9 (39.4)	0.364
Triglyceride, mg/dL, median (IQR)	103.6 (77.9)	100.9 (79.7)	107.1 (82.3)	103.5 (71.7)	100.9 (82.3)	100.0 (81.4)	0.142
High-density lipoprotein, mg/dL, mean (SD)	51.3 (15.3)	50.8 (15.0)	50.4 (15.5)	52.3 (15.6)	52.0 (15.2)	51.8 (14.5)	0.017
Low-density lipoprotein, mg/dL, mean (SD)	115.8 (34.1)	115.6 (32.9)	116.4 (34.5)	115.4 (33.8)	114.7 (34.1)	117.1 (36.9)	0.802
hs-CRP, mg/L, median (IQR)	1.0 (1.4)	0.9 (1.5)	1.0 (1.4)	1.0 (1.4)	1.0 (1.6)	1.0 (1.4)	0.195
eGFR, mL/min/1.73 m^2^, mean (SD)	94.4 (14.0)	97.3 (14.5)	93.8 (13.6)	93.6 (14.4)	93.4 (13.3)	94.5 (13.3)	< 0.001

*Abbreviations*: *ACEs* Adverse childhood experiences, *SD* Standard deviation, *IQR* Interquartile range (75th quartile minus 25th quartile), *BMI* Body mass index, *hs-CRP* High-sensitivity C-reactive protein, *eGFR* Estimated glomerular filtration rate

^a^*P* value was based on the chi-square test for categorical data and analysis of variance or the Kruskal–Wallis test for continuous data where appropriate

^b^Missing data: 1 for the area of residence, 4 for educational level, 194 for smoking, 42 for drinking, 89 for diabetes, 70 for hypertension, 144 for dyslipidemia, 45 for kidney,38 for lipid-lowering therapy, 100 for BMI, 98 for systolic pressure, 91 for diastolic pressure, 69 for depressive symptoms scores

^c^The depressive symptoms score was measured by the 10-item Center for Epidemiology Scale for Depression, ranging from 0 to 30, with higher scores indicating a higher level of depressive symptoms severity

^d^Measured in the subpopulation of 4113 participants

^e^Using Fisher’s exact test in r × c contingency tables

### Associations of ACEs and AAEs with incident CVD

A total of 789 participants experienced incident CVD (heart disease, 475 cases; stroke, 369 cases) during the follow-up period. The unadjusted incidence rates of incident CVD were higher among individuals exposed to ACEs or AAEs. After adjusting for covariates in model 4, an increased risk of incident CVD was associated with the overall number of ACEs (HR: 1.11, 95% CI: 1.08 to 1.14) and AAEs (HR: 1.19, 95% CI: 1.16 to 1.22), as shown in Table 2. A dose–response relationship between the number of ACEs or AAEs and incident CVD risk was observed, and the relationship remained significant in the fully adjusted model 4 (e.g., ACE [HR: 1.29, 95% CI:1.17 to 1.42 for one ACE indicator; HR: 1.39, 95% CI: 1.25 to 1.54 for two ACEs indicators; HR: 1.56, 95% CI: 1.40 to 1.75 for three indicators; HR: 1.58, 95% CI: 1.38 to 1.81 for at least four indicators]). For CVD components, even after adjusting for covariates in model 4, the overall number of ACEs and AAEs were associated with an increased risk of incident heart disease (ACE, HR: 1.06, 95% CI: 1.02 to 1.10; AAE, HR: 1.19, 95% CI: 1.15 to 1.24) and incident stroke (ACE, HR: 1.17, 95% CI: 1.13 to 1.20; AAE, HR: 1.17, 95% CI: 1.13 to 1.21). Similar dose–response relationships between the number of AAEs and the risks of incident heart disease or incident stroke were observed (Table 2).

**Table 2:** Incidence of cardiovascular disease (CVD) and hazard ratios for associations of adverse childhood experiences (ACEs) and adverse adulthood experiences (AAEs) with incident CVD

	**Case, No**	**Incidence rate, per 1000 person-years**	**HR (95% CI)**			
			**Model 1**	**Model 2**	**Model 3**	**Model 4**
**Cardiovascular disease**						
ACEs indicators^a^ (1-unit per increasing)	NA	NA	1.12 (1.09–1.14)	1.14 (1.11–1.17)	1.12 (1.05–1.20)	1.11 (1.08–1.14)
No. of ACEs indicators						
0	109	24.41	1.00 (reference)	1.00 (reference)	1.00 (reference)	1.00 (reference)
1	272	33.14	1.24 (1.13–1.35)	1.27 (1.16–1.39)	1.29 (1.17–1.42)	1.29 (1.17–1.42)
2	204	37.78	1.37 (1.25–1.51)	1.45 (1.32–1.60)	1.39 (1.25–1.54)	1.39 (1.25–1.54)
3	137	42.91	1.54 (1.39–1.71)	1.64 (1.48–1.82)	1.56 (1.39–1.74)	1.56 (1.40–1.75)
≥ 4	67	43.93	1.55 (1.37–1.76)	1.70 (1.50–1.93)	1.57 (1.37–1.80)	1.58 (1.38–1.81)
AAEs indicators^a^ (1-unit per increasing)	NA	NA	1.21 (1.18–1.24)	1.22 (1.19–1.25)	1.18 (1.15–1.22)	1.19 (1.16–1.22)
No. of AAEs indicators						
0	427	29.31	1.00 (reference)	1.00 (reference)	1.00 (reference)	1.00 (reference)
1	163	36.57	1.11 (1.03–1.19)	1.16 (1.07–1.25)	1.10 (1.01–1.19)	1.10 (1.01–1.19)
2	96	48.07	1.48 (1.35–1.62)	1.52 (1.38–1.66)	1.42 (1.29–1.56)	1.42 (1.29–1.56)
3	74	57.19	1.90 (1.71–2.10)	1.94 (1.75–2.15)	1.66 (1.49–1.85)	1.67 (1.50–1.86)
≥ 4	29	60.80	2.00 (1.71–2.33)	2.07 (1.77–2.42)	1.99 (1.70–2.34)	2.00 (1.70–2.35)
**Heart disease**						
ACEs indicators^a^ (1-unit per increasing)	NA	NA	1.08 (1.04–1.11)	1.10 (1.06–1.14)	1.06 (1.02–1.10)	1.06 (1.02–1.10)
No. of ACEs indicators						
0	62	13.88	1.00 (reference)	1.00 (reference)	1.00 (reference)	1.00 (reference)
1	156	19.01	1.25 (1.10–1.43)	1.30 (1.14–1.48)	1.33 (1.15–1.54)	1.33 (1.15–1.54)
2	129	23.89	1.24 (1.08–1.43)	1.31 (1.14–1.51)	1.25 (1.07–1.46)	1.25 (1.07–1.46)
3	85	26.62	1.39 (1.20–1.62)	1.49 (1.28–1.74)	1.42 (1.20–1.68)	1.42 (1.20–1.67)
≥ 4	43	28.20	1.41 (1.17–1.70)	1.55 (1.29–1.87)	1.37 (1.12–1.68)	1.37 (1.12–1.68)
AAEs indicators^a^ (1-unit per increasing)	NA	NA	1.23 (1.19–1.28)	1.24 (1.20–1.29)	1.19 (1.15–1.24)	1.19 (1.15–1.24)
No. of AAEs indicators						
0	265	18.19	1.00 (reference)	1.00 (reference)	1.00 (reference)	1.00 (reference)
1	96	21.54	1.20 (1.08–1.34)	1.25 (1.12–1.40)	1.17 (1.04–1.32)	1.17 (1.04–1.32)
2	53	26.54	1.60 (1.40–1.82)	1.63 (1.43–1.86)	1.57 (1.36–1.80)	1.57 (1.36–1.80)
3	42	32.46	1.91 (1.65–2.22)	1.96 (1.69–2.28)	1.55 (1.32–1.83)	1.55 (1.31–1.83)
≥ 4	19	39.83	2.13 (1.71–2.66)	2.19 (1.75–2.74)	2.04 (1.61–2.59)	2.04 (1.61–2.58)
**Stroke**						
ACEs indicators^a^ (1-unit per increasing)	NA	NA	1.16 (1.13–1.19)	1.18 (1.15–1.22)	1.16 (1.13–1.20)	1.17 (1.13–1.20)
No. of ACEs indicators						
0	52	11.86	1.00 (reference)	1.00 (reference)	1.00 (reference)	1.00 (reference)
1	135	16.83	1.27 (1.13–1.44)	1.31 (1.16–1.48)	1.32 (1.16–1.50)	1.32 (1.16–1.50)
2	91	17.26	1.56 (1.38–1.77)	1.66 (1.46–1.89)	1.60 (1.40–1.83)	1.60 (1.40–1.83)
3	61	19.70	1.72 (1.50–1.97)	1.84 (1.60–2.11)	1.76 (1.52–2.04)	1.76 (1.53–2.04)
≥ 4	30	20.30	1.79 (1.53–2.10)	1.98 (1.68–2.33)	1.86 (1.57–2.21)	1.87 (1.58–2.22)
AAEs indicators^a^ (1-unit per increasing)	NA	NA	1.19 (1.15–1.23)	1.20 (1.16–1.24)	1.17 (1.13–1.21)	1.17 (1.13–1.21)
No. of AAEs indicators						
0	192	13.45	1.00 (reference)	1.00 (reference)	1.00 (reference)	1.00 (reference)
1	81	18.59	1.06 (0.97–1.17)	1.12 (1.01–1.23)	1.06 (0.95–1.17)	1.06 (0.95–1.17)
2	48	24.64	1.34 (1.18–1.51)	1.37 (1.21–1.55)	1.25 (1.10–1.42)	1.25 (1.10–1.42)
3	34	27.35	1.74 (1.53–1.99)	1.79 (1.57–2.05)	1.62 (1.41–1.87)	1.63 (1.42–1.88)
≥ 4	14	30.70	2.11 (1.74–2.55)	2.22 (1.83–2.69)	2.17 (1.78–2.64)	2.17 (1.78–2.65)

Model 1 was adjusted for age and sex

Model 2 was adjusted for age, sex, residence, educational level, marital status, physical activity, smoking status, and alcohol consumption

Model 3 was adjusted as Model 2 plus body mass index; history of diabetes, hypertension, dyslipidemia, and chronic kidney disease; use of diabetes medications, hypertension medications, and lipid-lowering therapy; and depressive symptoms score

Model 4 was adjusted as Model 3 plus young adulthood social support

*Abbreviations*: *ACEs* Adverse childhood experiences, *AAEs* Adverse adulthood experiences, *No.* Number, *HR* Hazard ratio, *95% CI*, 95% confidence interval, *NA* Not available or not applicable

^a^Continuous variable

### Mediation analysis of AAEs on associations between ACEs and incident CVD

After adjusting for AAEs and other covariates, each additional ACE indicator was associated with a 9% (95% CI: 3 to 16%) higher risk of incident CVD, and the hazard ratios were larger without adjusting for AAEs. The overall AAEs were found to mediate 17.7% (95% CI: 8.2 to 34.2%) of the association between ACEs and incident CVD (Table 3). In addition, after adjusting for covariates other than AAEs, the adjusted HRs for incident CVD in individuals exposed to at least one ACE indicator were 1.38 (95 CI: 1.10 to 1.68) compared to those not exposed. However, after adjusting for AAEs and other covariates, the above associations decreased slightly to 1.36 (1.10 to 1.68), with the mediation proportion attributed to AAEs being 4.6% (95% CI: 1.3 to 15.4%) of the relationship (Table 3). Furthermore, the additional inclusion of AAEs did not enhance the predictive power of ACEs for incident CVD, shown in Additional file 2: Tab. S3.

**Table 3:** Associations of adverse childhood experiences (ACEs) with incident cardiovascular disease (CVD) and mediation proportion of the associations attributed to adverse adulthood experiences (AAEs)

	HR (95% CI)^a^		Mediation proportion (%) (95% CI)
	Unadjusted for AAEs	Adjusted for AAEs	
**Cardiovascular disease**			
ACEs indicators^b^ (1-unit per increasing)	1.11 (1.08–1.14)	1.09 (1.03–1.16)	17.7 (8.2–34.2)
No. of ACEs indicators			
0	1.00 (reference)	1.00 (reference)	-
≥ 1	1.38 (1.12–1.70)	1.36 (1.10–1.68)	4.6 (1.3–15.4)
**Heart disease**			
ACEs indicators^b^ (1-unit per increasing)	1.06 (1.02–1.10)	1.04 (0.98–1.08)	36.7 (4.0–89.0)
No. of ACEs indicators			
0	1.00 (reference)	1.00 (reference)	-
≥ 1	1.32 (1.16–1.51)	1.30 (1.14–1.49)	5.6 (1.0–26.1)
**Stroke**			
ACEs indicators^b^ (1-unit per increasing)	1.17 (1.13–1.20)	1.15 (1.11–1.18)	10.9 (4.5–24.0)
No. of ACEs indicators			
0	1.00 (reference)	1.00 (reference)	-
≥ 1	1.51 (1.34–1.70)	1.49 (1.32–1.68)	3.3 (0.9–11.9)

*Abbreviations*: *ACEs* Adverse childhood experiences, *AAEs* Adverse adulthood experiences, *HR* Hazard ratio, *95% CI*, 95% confidence interval

^a^Models were adjusted for age, sex, residence, marital status, educational level, health behaviors (physical activity, smoking status, and alcohol consumption), health status variables (BMI; history of diabetes, hypertension, dyslipidemia, and chronic kidney disease; use of diabetes medications, hypertension medications, and lipid-lowering therapy; and depressive symptoms), and young adulthood social support

^b^Continuous variable

For CVD components, after adjusting for AAEs and other covariates, the associations of the number of ACE indicators with incident heart disease and incident stroke were slightly decreasing compared to the observed associations from the models without adjusting for AAEs. The mediation proportion attributed to AAEs was 36.7% (95% CI: 4.0 to 89.0%) for the associations between the overall ACEs and incident heart disease and 10.9% (95% CI: 4.5 to 24.0%) for the associations for incident stroke, respectively (Table 3). Moreover, the additional inclusion of AAEs did not enhance the predictive power of ACEs for incident heart disease or incident stroke, shown in Additional file 2: Tab. S3.

### Interaction and joint analysis of ACEs and AAEs on incident CVD

**Fig. 2:**
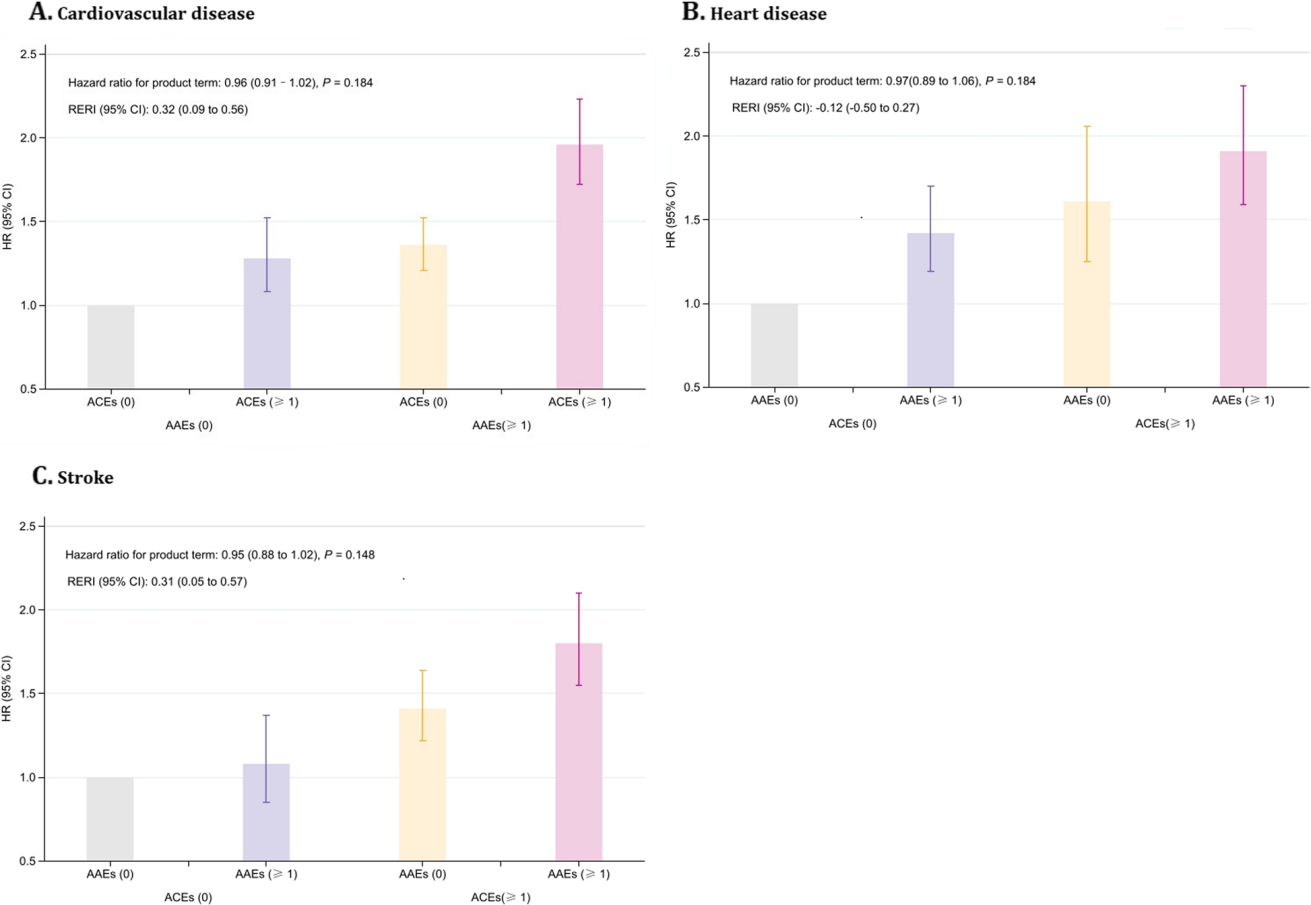
Interaction and joint analysis of adverse childhood experiences and adverse adulthood experiences with incident cardiovascular disease

In the fully adjusted model, no significant multiplicative interaction was observed between ACEs and AAEs on incident CVD (*P* = 0.184, Fig. 2A), but a significant additive interaction was detected (RERI [95% CI]: 0.32 [0.09 to 0.56], Fig. 2A). The joint analysis of ACEs and AAEs on incident CVD revealed that compared to those not exposed to either, the adjusted HR for adults exposed to at least one ACE and one AAE indicator was highest at 1.96 (95% CI: 1.72 to 2.23) (Fig, 2A).

For CVD components, in the fully adjusted model, no significant multiplicative interaction was observed between ACEs and AAEs on incident heart disease (*P* = 0.184, Fig. 2B) and incident stroke (*P* = 0.148, Fig. 2C), but a significant additive interaction was observed for incident stroke (RERI [95% CI]: 0.31 [0.05 to 0.57], Fig. 2C). The joint analysis for incident heart disease or incident stroke demonstrated that exposure to at least one ACE and one AAE indicator was associated with the highest magnitude of both incident heart disease (HR: 1.91, 95% CI: 1.59 to 2.30) and incident stroke (HR: 1.80, 95% CI: 1.55 to 2.10) (Fig. 2B and C).

### Modifying roles of social support, biological sex, and age

As shown in Additional file 2: Tab. S4, young adulthood social support was a significant modifier in the associations of ACEs or AAEs with incident CVD (ACEs × social support: *β* [95% CI], − 0.05 [− 0.09 to − 0.02]; AAEs × social support: *β* [95% CI], − 0.04 [− 0.08 to − 0.01]) in the age- and sex-adjusted model (model 1). However, after adjusting for other covariates, these significant associations disappeared in models 2 and 3. The role of biological sex as a modifier in the associations of ACEs or AAEs with incident CVD was not significant, regardless of whether covariates were included or not. On the other hand, age was found to be a significant modifier in the associations of ACEs or AAEs with incident CVD in model 3 (ACEs × age: *β* [95% CI], − 0.01 [− 0.01 to − 0.01]; AAEs × age: *β* [95% CI], 0.003 [0.001 to 0.01]).

Regarding CVD components, the modifying role of social support during young adulthood was observed in the association between AAEs and incident heart disease (AAEs × social support: *β* [95% CI], − 0.08 [− 0.13 to − 0.02]). Additionally, the modifying roles of biological sex and age were observed in the association between ACEs and incident heart disease (ACEs × sex: *β* [95% CI], 0.09 [0.01 to 0.17]); ACEs × age: *β* [95% CI], − 0.01 [− 0.01 to − 0.01]). Furthermore, age was found to be a significant modifier in the associations of both ACEs and AAEs with incident stroke (ACEs × age: *β* [95% CI], − 0.01 [− 0.01 to − 0.01]; AAEs × age: *β* [95% CI], 0.01 [0.001 to 0.01]), even adjusting for covariates in model 3.

### Subgroup analyses

The results stratified by sex and age group are presented in Additional file 2: Tab. S5–Tab. S6 and Additional file 3: Fig. S2–Fig. S3. After adjusting for covariates, the associations of ACEs or AAEs with incident CVD did not differ significantly between males and females, but the negative association between young adulthood social support and incident CVD was statistically significant only in females. The associations of ACEs with incident CVD were significant only in adults under 60 years old, while the associations of AAEs with incident CVD were stronger in older compared to younger adults, shown in Additional file 2: Tab. S5. The proportions that AAEs mediated in the association between ACEs and incident CVD were similar to the main findings in both males and females and younger adults. However, AAEs did not mediate the association between ACEs and incident CVD among older adults, shown in Additional file 2: Tab. S6. The results of the joint analyses of ACEs and AAEs with incident CVD were consistent with the primary findings, except for significant multiplicative and additive interactions between ACEs and AAEs on incident CVD observed among adults aged < 60 years, shown in Additional file 3: Fig. S3.

### Sensitivity analyses

The associations of ACEs or AAEs with incident CVD did not significantly change after further adjusting for fasting plasma glucose, glycosylated hemoglobin, total cholesterol, triglyceride, high-density lipoprotein, low-density lipoprotein, hs-CRP, and eGFR, shown in Additional file 2: Tab. S7. Similar findings were obtained from complete data analyses, shown in Additional file 2: Tab. S8–Tab. S9, except for the absence of a significant additive interaction, shown in Additional file 3: Fig. S4. The fully adjusted models revealed that six types of ACEs indicators (including childhood physical neglect, childhood domestic violence, childhood parental death, childhood physical abuse, childhood household mental illness, and childhood exposure to natural disasters) and three types of AAEs indicators (including ever being confined to bed in adulthood, ever being hospitalized for a month or longer, and ever leaving a job due to health conditions) were significantly associated with incident CVD. Moreover, childhood domestic violence, childhood exposure to natural disasters, and childhood physical abuse were the 3 most prominent ACEs associated with incident CVD, shown in Additional file 2: Tab. S10. The mediation proportion by the overall AAEs for the associations between each ACE indicator and incident CVD showed similar patterns, shown in Additional file 2: Tab. S11.

## Discussion

In this cohort study of middle-aged or older adults in China, exposure to ACEs or AAEs was independently associated with an increased risk of incident CVD, and the risk of incident CVD was found to increase with an increasing number of ACE or AAE indicators. Furthermore, the association between ACEs and incident CVD was partially explained by AAEs. An additive interaction was observed between ACEs and AAEs on incident CVD, with the highest risks seen in adults exposed to at least one ACE and one AAE indicator.

Despite consistent reports linking ACEs with CVD in various cultural contexts [5–7], there is limited research investigating the impacts of both ACEs and AAEs on incident CVD, while also considering a series of covariates, including social support. Moreover, previous studies have shown associations between ACEs and CVD risk factors such as smoking, alcohol consumption [8, 42], and depression [11, 12], but there has been a lack of focus on their confounding influences in the associations between ACEs and CVD events. After adjusting for several potential confounding variables, including CVD risk factors and social support, this study still found positive associations of ACEs and AAEs with incident CVD, with a dose–response relationship between the number of ACEs or AAEs and increased risks for both males and females. Similar positive relationships were observed between the number of ACEs or AAEs and the risks of incident heart disease and incident stroke, which are components of the investigated CVD events. These findings align with a previous retrospective cohort study using the UK biobank, which demonstrated a dose–response relationship between the number of childhood maltreatment types and incident CVD [11]. A cross-sectional study in German adults also supports these results, revealing a significant and independent association between adulthood stressful life events and cardiovascular health [16]. The present study expands on previous research by demonstrating the associations of both ACEs and AAEs with incident CVD and its subgroups (i.e., heart disease and stroke) while controlling for potential CVD risk factors and young adulthood social support. Although the potential use of interventions focused on ACE or AAE for cardiovascular health has not been tested in large randomized clinical trials, the potential reversibility of ACE or AAE consequences in adults has received attention [5]. Our findings may provide insight into the early identification of vulnerable groups and the prioritization of prevention and intervention efforts for individuals with ACE or AAE exposure to safeguard their cardiovascular health. Among the individual ACEs examined, childhood domestic violence exhibited the strongest association with incident CVD, followed by childhood exposure to natural disasters and childhood physical abuse. These specific ACEs appear to be crucial aspects that need to be considered in ACE prevention strategies.

The exact mechanism underlying the associations of ACEs or AAEs with incident CVD remains poorly understood. Dysregulation of the hypothalamic–pituitary–adrenal (HPA) axis, chronic inflammatory processes, increased cardiac electrical instability, myocardial ischemia, and functional changes in the central nervous system have been suggested as potential pathophysiological effects of adverse experiences in childhood or adulthood on the development and progression of CVD events [42, 43]. In addition to the mechanisms mentioned above, previous evidence also suggests that ACEs or AAEs may predispose individuals to poor mental health and an unhealthy lifestyle, including hypertension, high serum cholesterol levels, diabetes, smoking, and obesity, which in turn, predispose individuals to CVD [5, 11, 12]. Therefore, our findings regarding the associations of ACEs or AAEs with incident CVD are physiologically plausible. Nevertheless, further evidence is needed to elucidate the underlying mechanism fully.

Prior evidence suggests AAEs may play a crucial role as a disease trigger in the relationship between ACEs and adult health [15]. The present study builds upon these findings by demonstrating the mediating role of AAEs in the association between ACEs and incident CVD. Our results indicate that the association between ACEs and incident CVD was partly explained by AAEs, highlighting the importance of this pathway in the etiology of CVD. The stress generation model may explain the mediating role of AAEs [44]. Specifically, early life stress may increase the likelihood of encountering more significant stressors in adulthood, with subsequent exposure largely responsible for any variance linked to ACEs [14, 44]. Notably, ACEs, such as neglect and abuse, may inculcate cognitive biases toward the threat, thereby fostering a heightened sense of danger, even in ambiguous circumstances [45]. These negative perceptions may increase anxiety and stress levels over the lifespan. Stressful experiences in adulthood, such as job strain and discrimination, have been linked to poorer overall health and a higher prevalence of conditions such as hypertension and stroke [16]. Importantly, our findings also provide evidence of the significance of avoiding AAEs, which may help mitigate the association between ACEs and incident CVD.

Furthermore, this study observed a significant additive interaction effect between ACEs and AAEs on incident CVD. Notably, the risk associations of AAEs with incident CVD were stronger among those exposed to at least one ACE indicator, highlighting the critical importance of preventing adversity experiences for adults, particularly among those who are more vulnerable to AAEs due to prior exposure to ACEs. These results align with a longitudinal analysis in the Finnish Public Sector, which revealed that the combined effects of childhood psychosocial adversity and adult neighborhood disadvantage increased the risk of CVD [7]. However, a separate longitudinal study in the USA found that ACEs and recent life events were independently associated with a higher level of inflammation at midlife, with no evidence of synergistic effects. Notably, elevated inflammation levels have been shown to be related to CVD [15]. Therefore, given the mixed results, these findings offer valuable insights, but definitive conclusions about the interaction effects between ACEs and AAEs on CVD cannot be drawn. Further research is needed to fully elucidate the complex relations between ACEs and AAEs on the development of CVD.

Previous evidence suggests that early life social support may facilitate the development of effective coping and emotion regulation strategies to mitigate the impact of childhood and adulthood stress, as well as reduce CVD risk factors associated with ACEs or AAEs [19]. However, our study only revealed mild buffering effects of social support during young adulthood on the negative consequences of ACEs or AAEs for CVD. Further research is needed to investigate the role of social support across different age groups, including the influence of additional forms of support such as family and friend support. It is also important to explore additional significant factors that can modify the association between ACEs or AAEs and CVD. Our findings also demonstrated that age could significantly modify the relationship between ACEs or AAEs and incident CVD. Regarding CVD components, biological sex, and age may act as modifiers in the association between ACEs and incident heart disease, while age may play a modifying role in the associations between AAEs and incident stroke. Further subgroup analysis revealed that the associations of ACEs or AAEs with incident CVD did not significantly differ between males and females. However, the associations of ACEs with incident CVD were significant only in adults under 60 years old, whereas the associations of AAEs with incident CVD were stronger in older adults compared to younger ones. Among older adults (> 60 years old), the mediating role of AAEs disappeared in the association between ACEs and incident CVD, suggesting the existence of alternative pathways linking ACEs to later health outcomes [15] and the impact of ACEs on stress reactivity may diminish with age. However, this finding could also be attributed to the fact that individuals with cardiovascular events resulting from ACEs or AAEs may have already passed away by the age of 60. Nevertheless, these findings emphasize the need for further investigation into the modifying roles of biological sex and age.

The strengths of our study include the cohort design and large sample size, which provided ample statistical power to explore the associations of ACEs and AAEs with incident CVD, while enabling mediation and subgroup analyses. Furthermore, this study comprehensively estimated the impacts of both ACEs and AAEs on subsequent CVD events, with consideration of a series of covariates including young adulthood social support. However, several limitations are worth noting. First, the information on ACEs and AAEs relied on retrospective self-reports, which may have been vulnerable to reporting and recall bias. Nevertheless, previous research has demonstrated good test–retest reliability of retrospective measurements of ACEs [25]. Second, the main analysis only summed up the cumulative numbers of ACEs or AAEs, which assumes that different types of ACEs or AAEs have equivalent effects on CVD, possibly disregarding unique information. Nonetheless, this study conducted sensitivity analyses utilizing each ACE or AAE indicator, yielding similar mediation proportion patterns. Third, the study used self-reported doctors’ diagnoses, which may have underestimated the occurrence of CVD events. Moreover, the specific data on categories of heart disease, such as heart attack and coronary heart disease, were not available for our analyses. Fourth, residual confounding remains possible despite controlling for multidimensional covariates, including demographic characteristics, health behavior factors, health status factors, young adulthood social support, and metabolic biomarkers, and causal relationships cannot be confirmed. Fifth, the study findings need to be validated by other cohort studies and randomized control trials. Sixth, although previous studies using the CHARLS dataset reported slightly lower values about the number of CVD cases/total sample size than the present study [3, 18], the low number of CVD cases/total sample size may be a concern. Seventh, it is important to note that the CHARLS dataset only provides data on young adulthood social support. However, social support across other age groups and other forms of support, such as family support and friend support, may also play a role in the associations between ACEs and AAEs with incident CVD. Eighth, in this study, the difference method was used instead of structural equation modeling to estimate the proportion of mediation by the overall AAEs in the association between ACEs and incident CVD. However, conducting specific mediation/path analysis using structural equation modeling could examine direct and indirect estimates. Moreover, the confidence intervals for the mediations have wide ranges, and further research is necessary to validate the study results regarding the mediation of AAEs on the associations between ACEs and incident CVD.

## Conclusions

In summary, exposure to ACEs or AAEs could be associated with an increased risk of CVD among middle-aged and older adults in a dose–response manner. We also observed a modest mediation of AAEs on the association between ACEs and incident CVD. Notably, we found a significant additive interaction effect between ACEs and AAEs on the risk of incident CVD, with individuals exposed to both stressors exhibiting the highest risk. Accordingly, the findings suggest that preventing ACEs alone might not substantially reduce the risk of incident CVD later in life, and other strategies for tackling AAEs may also be needed. Therefore, a comprehensive life-course health strategy targeting the prevention of both childhood and adulthood stressors may have potential value in mitigating the risks of incident CVD. However, future randomized clinical trials are needed to confirm these conclusions.

### Supplementary Information

**Additional file 1: Appendix. S1.** Study population and definition of covariates in detail.

**Additional file 2: Table S1.** Question items and responses for variables included in the adverse childhood and adulthood experiences; **Table S2.** Baseline characteristics of the study population by the number of adverse adulthood experiences (AAEs); **Table S3.** C-statistics of models without and with adverse adulthood experiences (AAEs); **Table S4.** Young adulthood social support, sex, and age as effect modifiers for the associations of adverse childhood experiences (ACEs) and adverse adulthood experiences (AAEs) with incident cardiovascular disease (CVD); **Table S5.** Hazard ratios for associations of adverse childhood experiences (ACEs), adverse adulthood experience (AAEs), and young adulthood social support with incident cardiovascular disease (CVD): subgroup analyses; **Table S6.** Associations of adverse childhood experiences (ACEs) with incident cardiovascular disease (CVD) and mediation proportion of the associations attributed to adverse adulthood experience (AAEs): subgroup analyses; **Table S7.** Associations of adverse childhood experience (ACEs) and adverse adulthood experiences (AAEs) with incident cardiovascular disease (CVD) in the subpopulation of 4113 participants with metabolic biomarkers measurements; **Table S8.** Hazard ratios for associations of adverse childhood experiences (ACEs), adverse adulthood experiences (AAEs), and young adulthood social support with incident cardiovascular disease (CVD) using the complete dataset; **Table S9.** Associations of adverse childhood experiences (ACEs) with incident cardiovascular disease (CVD) and mediation proportion of the associations attributed to adverse adulthood experiences (AAEs): using the complete dataset; **Table S10.** Hazard ratios for associations of each indicator of adverse childhood experiences (ACEs) and adverse adulthood experiences (AAEs) with incident cardiovascular disease (CVD); **Table S11.** Associations of each indicator of adverse childhood experiences (ACEs) with incident cardiovascular disease (CVD) and mediation proportion of the associations attributed to adverse adulthood experiences (AAEs).

**Additional file 3: Figure S1.** Overlapping exposure to adverse childhood experiences (ACEs) and adverse adulthood experiences (AAEs); **Figure 2.** Interaction and joint analysis of adverse childhood experiences (ACEs) and adverse adulthood experience (AAEs) with incident cardiovascular disease (CVD): subgroup analysis by sex; **Figure S3.** Interaction and joint analysis of adverse childhood experiences (ACEs) and adverse adulthood experience (AAEs) with incident cardiovascular disease (CVD): subgroup analyses: subgroup analysis by age; **Figure S4.** Interaction and joint analysis of adverse childhood experiences (ACEs) and adverse adulthood experience (AAEs) with incident CVD: using the complete dataset.

## Data Availability

The data that support the findings of the study are available through the website of CHARLS: http://charls.pku.edu.cn/index/en.html. To access and use the day for research purpose, approval should be obtained from the CHARLS team at Peking University.

## Abbreviations

AAEs: Adverse adulthood experiences
ACEs: Adverse childhood experiences
AHA: American Heart Association
BMI: Body mass index
CHARLS: China Health and Retirement Longitudinal Study
CVD: Cardiovascular disease
eGFR: Estimated glomerular filtration rate
HbA1c: Glycosylated hemoglobin
Hs-CRP: High-sensitivity C-reactive protein
