# Supplementary material for: Adverse childhood and adulthood experiences and risk of new-onset cardiovascular disease with consideration of social support: a prospective cohort study
Source: BMC Med. 2023 Aug 8;21:297. doi: 10.1186/s12916-023-03015-1 (PMC10408183; doi:10.1186/s12916-023-03015-1)
Supplement: Supplementary file 1 — Additional file 1: Appendix. S1. Study population and definition of covariates in detail. [file 12916_2023_3015_MOESM1_ESM.docx]

**Appendix. S1.** Study population and definition of covariates in detail.

*Study population*

The data for this study were obtained from the 2015 and 2018 main surveys of the Chinese Health and Retirement Longitudinal Study (CHARLS). A total of 20967 participants were initially recruited in the CHARLS 2015 survey. Among these individuals, we retrospectively matched 18718 participants in a 1:1 ratio with the 2014 life course survey, using community ID, household ID, and individual ID simultaneously. Based on pre-defined inclusion and exclusion criteria, we excluded 10421 individuals without the required information on any ACE or AAE indicator measures, 132 individuals aged below 45 years or without age information, 1050 individuals with CVD in the CHARLS 2015 survey, and 1279 individuals with no CVD data in the CHARLS 2018 survey. The final cohort for analysis included 5836 individuals.

*Definition of covariates*

Physical activity was assessed based on whether the participant engaged in vigorous activity ≥ 3 times per week (responses coded as yes or no). Body mass index (BMI) was calculated as the ratio of body weight in kilograms to height in meters squared (BMI = kg/m^2^).

Depressive symptoms were evaluated using the 10-item Center for Epidemiology Scale for Depression (CESD-10), which has been validated and widely used among Chinese adults. The CESD-10 consists of ten items: (1) bothered by little things, (2) had trouble concentrating, (3) felt depressed, (4) everything was an effort, (5) felt hopeful, (6) felt fearful, (7) sleep was restless, (8) felt happy, (9) felt lonely, and (10) could not get going. Responses for each depressive symptom item in the past week were measured on a scale from 0 (rarely or none of the time [<1 day]) to 3 (most or all of the time [5-7 days]). The total score for the CESD-10 ranges from 0 to 30, with higher scores indicating more severe depressive symptoms. A cutoff score of 12 or higher was used to indicate the presence of depressive symptoms.
