# Supplementary material for: Adverse childhood and adulthood experiences and risk of new-onset cardiovascular disease with consideration of social support: a prospective cohort study
Source: BMC Med. 2023 Aug 8;21:297. doi: 10.1186/s12916-023-03015-1 (PMC10408183; doi:10.1186/s12916-023-03015-1)
Supplement: Supplementary file 2 — Additional file 2: Table S1. Question items and responses for variables included in the adverse childhood and adulthood experiences; Table S2. Baseline characteristics of the study population by the number of adverse adulthood experiences (AAEs); Table S3. C-statistics of models without and with adverse adulthood experiences (AAEs); Table S4. Young adulthood social support, sex, and age as effect modifiers for the associations of adverse childhood experiences (ACEs) and adverse adulthood experiences (AAEs) with incident cardiovascular disease (CVD); Table S5. Hazard ratios for associations of adverse childhood experiences (ACEs), adverse adulthood experience (AAEs), and young adulthood social support with incident cardiovascular disease (CVD): subgroup analyses; Table S6. Associations of adverse childhood experiences (ACEs) with incident cardiovascular disease (CVD) and mediation proportion of the associations attributed to adverse adulthood experience (AAEs): subgroup analyses; Table S7. Associations of adverse childhood experience (ACEs) and adverse adulthood experiences (AAEs) with incident cardiovascular disease (CVD) in the subpopulation of 4113 participants with metabolic biomarkers measurements; Table S8. Hazard ratios for associations of adverse childhood experiences (ACEs), adverse adulthood experiences (AAEs), and young adulthood social support with incident cardiovascular disease (CVD) using the complete dataset; Table S9. Associations of adverse childhood experiences (ACEs) with incident cardiovascular disease (CVD) and mediation proportion of the associations attributed to adverse adulthood experiences (AAEs): using the complete dataset; Table S10. Hazard ratios for associations of each indicator of adverse childhood experiences (ACEs) and adverse adulthood experiences (AAEs) with incident cardiovascular disease (CVD); Table S11. Associations of each indicator of adverse childhood experiences (ACEs) with incident cardiovascular disease (CVD) and mediati [file 12916_2023_3015_MOESM2_ESM.docx]

|  | | | |
| --- | --- | --- | --- |
| **Table S1.** Question items and responses for variables included in the adverse childhood and adulthood experiences. | | | |
| **Categories** | | **Questionnaire items** | **N (%)** |
| **Adverse childhood experiences (ACEs)** | | | |
| Household dysfunction | Household substance use | During the years you were growing up, did your female/male guardian ever have alcoholism or drug? (Responses were categorized as 0=no and 1=yes). | 457 (7.8) |
|  | Household mental illness | Did your female/male guardian have an abnormality of mind when you were young? (Responses were categorized as 0=no and 1=yes). | 1369 (23.5) |
|  | Domestic violence | Have your father/mother ever beat up your mother/father? (Responses were categorized to 0=not very often or never; 1=often or sometimes). | 492 (8.4) |
|  | Criminal behavior in the household | During the years you were growing up, have your female/male guardian ever been involved in criminal activities like burglary or selling stolen property, or arrested or sent to prison? (Responses were categorized as 0=no and 1=yes). | 21 (0.4) |
|  | Parental separation or divorce | Were your biological parents divorced (including a long separation due to emotional problems) before you were 17 years old? | 27 (0.5) |
|  | Parental death | Either of the parents was dead before the participant was 17 years old. (The answer was calculated based on the date of birth and their parental death, and responses were categorized to 0=no and 1=yes). | 1132 (19.4) |
| Neglect and abuse | Physical neglect | When you were a child before age 17, was there ever a time when your family did not have enough food to eat? (Responses were categorized as 0=no and 1=yes). | 4115 (70.9) |
|  | Emotional neglect | How much love and affection did your female guardian give you while you were growing up? (Responses were categorized as 0=often or sometimes; 1=rarely or never). | 1207 (20.9) |
|  | Physical abuse | When you were growing up, did your female/male guardian ever hit you? (Responses were categorized as 0=not very often or never; 1=often or sometimes). | 1197 (20.5) |
| Additional ACE | Exposure to natural disasters | Between 1958-1962 did you and your family (including your grandparents, parents, siblings, children and so on) experience starvation or move away from the famine-stricken area? (Responses were categorized as 0=no and 1=yes). | 450 (8.0) |
| **Table S1.** Question items and responses for variables included in the adverse childhood and adulthood experiences (continued). | | | |
| **Adverse adulthood experiences (AAEs)** | | | |
|  | Death of the child | Death of the participant’s child (Responses were categorized as 0=no and 1=yes). | 680 (11.7) |
|  | Experiencing lifetime discrimination | After you were 16 years old, because of ill health, did you experience any of the following (denied promotions, assignment to a task with fewer responsibilities, working on tasks below your qualifications, harassment by your boss or colleagues, pay cuts, dismissed)? (Responses were categorized as 0=no and 1=yes). | 574 (9.8) |
|  | Ever being confined to bed | After you were 16 years old, because of a health condition, were you ever confined to bed or home for one month or more? (Responses were categorized as 0=no and 1=yes). | 841 (14.4) |
|  | Ever being hospitalized for a month or longer | After you were 16 years old, because of a health condition, were you ever hospitalized for a month or more? (Responses were categorized as 0=no and 1=yes). | 632 (10.8) |
|  | Ever leaving a job due to health conditions | After you were 16 years old, because of a health condition, did you leave your job for one month or more? (Responses were categorized as 0=no and 1=yes). | 962 (16.5) |

Abbreviation: ACEs, adverse childhood experiences; AAEs, adverse adulthood experiences.

| **Table S2.** Baseline characteristics of the study population by the number of adverse adulthood experiences (AAEs). | | | | | | |
| --- | --- | --- | --- | --- | --- | --- |
| **Characteristics** | **Number of AAEs indicators, (N=5836)** | | | | | |
|  | **0 (n=3721)** | **1 (n=1140)** | **2 (n=516)** | **3 (n=334)** | **≥4 (n=125)** | ***P* value^a^** |
| Age, mean (SD), y | 58.7 (7.7) | 61.5 (8.9) | 61.7 (9.2) | 60.2 (8.2) | 59.9 (8.6) | <0.001 |
| Sex, n (%) |  |  |  |  |  |  |
| Male | 1819 (48.9) | 538 (47.2) | 286 (55.4) | 191 (57.2) | 67 (53.6) | 0.001 |
| Female | 1902 (51.1) | 602 (52.8) | 230 (44.6) | 143 (42.8) | 58 (46.4) |  |
| Residential area^b^, n (%) |  |  |  |  |  |  |
| Rural | 2984 (80.2) | 993 (87.1) | 425 (82.4) | 283 (84.7) | 114 (91.2) | <0.001 |
| Urban | 736 (19.8) | 147 (12.9) | 91 (17.6) | 51 (15.3) | 11 (8.8) |  |
| Marital status |  |  |  |  |  |  |
| Married | 3217 (86.5) | 946 (83.0) | 429 (83.1) | 279 (83.5) | 101 (80.8) | 0.009 |
| Other marital status | 504 (13.5) | 194 (17.0) | 87 (16.9) | 55 (16.5) | 24 (19.2) |  |
| Educational level^b^, n (%) |  |  |  |  |  |  |
| No formal education | 1303 (35.0) | 547 (48.0) | 225 (43.6) | 154 (46.1) | 59 (47.2) | <0.001^e^ |
| Primary school or below | 855 (23.0) | 278 (24.4) | 117 (22.7) | 70 (21.0) | 28 (22.4) |  |
| Middle or high school | 1471 (39.6) | 299 (26.3) | 165 (32.0) | 106 (31.7) | 38 (30.4) |  |
| College or above | 89 (2.4) | 15 (1.3) | 9 (1.7) | 4 (1.2) | 0 |  |
| Smoking status^b^, n (%) |  |  |  |  |  |  |
| Nonsmoker | 2278 (63.2) | 698 (63.0) | 269 (54.5) | 189 (59.4) | 75 (63.6) | 0.015 |
| Former smoker | 247 (6.9) | 71 (6.4) | 50 (10.1) | 24 (7.5) | 6 (5.1) |  |
| Current smoker | 1079 (29.9) | 339 (30.6) | 175 (35.4) | 105 (33.0) | 37 (31.4) |  |
| Alcohol consumption^b^, n (%) |  |  |  |  |  |  |
| Nondrinker | 2212 (59.8) | 653 (57.9) | 275 (53.6) | 194 (58.4) | 65 (53.3) | 0.229 |
| Former drinker | 323 (8.7) | 95 (8.4) | 50 (9.7) | 25 (7.5) | 11 (9.0) |  |
| Current drinker | 1165 (31.5) | 379 (33.6) | 188 (36.6) | 113 (34.0) | 46 (37.7) |  |
| Physical activity (≥ 3 × a week)^b^, n (%) |  |  |  |  |  |  |
| Yes | 3163 (85.0) | 941 (82.5) | 436 (84.5) | 289 (86.5) | 110 (88.0) | 0.182 |
| No | 558 (15.0) | 199 (17.5) | 80 (15.5) | 45 (13.5) | 15 (12.0) |  |
| Health conditions (yes)^b^, n (%) |  |  |  |  |  |  |
| Diabetes | 157 (4.3) | 42 (3.8) | 27 (5.3) | 27 (8.2) | 5 (4.2) | 0.010 |
| Hypertension | 650 (17.6) | 238 (21.3) | 101 (19.8) | 84 (25.3) | 21 (17.4) | 0.002 |
| Dyslipidemia | 264 (7.3) | 74 (6.7) | 35 (7.0) | 28 (8.6) | 6 (5.0) | 0.675 |
| Chronic kidney disease | 157 (4.2) | 79 (7.0) | 56 (10.9) | 30 (9.0) | 11 (9.1) | <0.001 |
| History of medication use (yes)^b^, n (%) |  |  |  |  |  |  |
| **Table S2.** Baseline characteristics of the study population by the number of adverse adulthood experiences (AAEs) (continued). | | | | | | |
| Diabetes medications | 105 (2.8) | 32 (2.8) | 19 (3.7) | 21 (6.3) | 4 (3.2) | 0.011 |
| Hypertension medications | 489 (13.1) | 169 (14.8) | 77 (14.9) | 60 (18.0) | 17 (13.6) | 0.105 |
| Lipid-lowering therapy | 137 (3.9) | 37 (3.5) | 19 (3.9) | 17 (5.4) | 3 (2.6) | 0.554 |
| BMI^b^, kg/m2, mean (SD) | 23.8 (4.0) | 23.15 (4.3) | 23.2 (4.2) | 23.4 (5.0) | 24.0 (5.0) | <0.001 |
| Blood pressure^b^, mm Hg, mean (SD) |  |  |  |  |  |  |
| Systolic pressure | 127.2 (21.1) | 129.8 (21.6) | 129.1 (20.1) | 129.6 (21.3) | 128.3 (19.3) | 0.016 |
| Diastolic pressure | 75.2 (11.9) | 75.2 (11.8) | 76.3 (11.5) | 75.0 (12.8) | 76.6 (11.5) | 0.383 |
| Depressive symptoms scores^c^, mean (SD) | 7.1 (5.7) | 8.6 (6.4) | 9.4 (6.6) | 9.7 (6.9) | 10.5 (6.1) | <0.001 |
| Adulthood social support, mean (SD) | 0.3 (0.7) | 0.3 (0.6) | 0.4 (0.7) | 0.4 (0.7) | 0.5 (0.8) | 0.004 |
| Metabolic biomarkers^d^ |  |  |  |  |  |  |
| Fasting plasma glucose, mg/dL mean (SD) | 109.4 (35.9) | 109.7 (32.6) | 106.1 (23.2) | 110.6 (35.1) | 104.0 (26.7) | 0.243 |
| HbA1c (%), mean (SD) | 5.2 (0.8) | 5.3 (0.8) | 5.2 (0.6) | 5.3 (0.8) | 5.2 (0.8) | 0.199 |
| Total cholesterol, mg/dL, mean (SD), | 192.0 (37.4) | 195.0 (37.9) | 193.1 (37.9) | 192.6 (36.6) | 186.2 (35.7) | 0.147 |
| Triglyceride, mg/dL, median (IQR) | 103.5 (78.8) | 104.4 (77.9) | 103.5 (74.3) | 103.5 (85.4) | 102.7 (70.8) | 0.928 |
| High-density lipoprotein, mg/dL, mean (SD) | 50.7 (14.8) | 52.8 (16.0) | 52.6 (16.6) | 50.7 (16.6) | 48.4 (12.8) | 0.003 |
| Low-density lipoprotein, mg/dL, mean (SD) | 116.0 (34.1) | 116.2 (34.6) | 115.0 (34.1) | 115.2 (33.3) | 111.1 (33.6) | 0.716 |
| hs-CRP, mg/L, median (IQR) | 0.9 (0.9) | 1.0 (1.6) | 1.1 (1.4) | 1.1 (1.6) | 1.0 (1.8) | 0.098 |
| eGFR, mL/min/1.73 m^2^, mean (SD) | 95.1 (13.5) | 92.6 (15.2) | 92.9 (14.1) | 95.0 (13.0) | 97.4 (14.7) | <0.001 |

Abbreviation: AAEs, adverse adulthood experiences; SD, standard deviation; IQR, interquartile range (75th quartile minus 25th quartile); BMI, body mass index; hs-CRP, high-sensitivity C-reactive protein; eGFR, estimated glomerular ﬁltration rate.

^a^ *P* value was based on the Chi-square test for categorical data and analysis of variance or the Kruskal-Wallis test for continuous data where appropriate.

^b^ *P* value for trend was based on the Mantel-Haenszel statistic for categorical data and analysis of variance in trends with polynomial comparisons or the Jonckheere-Terpstra test for continuous data where appropriate.

^c^ Missing data: 1 for the area of residence, 4 for educational level, 194 for smoking, 42 for drinking, 89 for diabetes, 70 for hypertension, 144 for dyslipidemia, 45 for kidney,38 for lipid-lowering therapy, 100 for BMI, 98 for systolic pressure, 91 for diastolic pressure, 69 for depressive symptoms scores.

^d^ The depressive symptoms scores were measured by the 10-item Center for Epidemiology Scale for Depression, ranging from 0 to 30, with higher scores indicating a higher level of depressive symptoms severity.

^e^ Measured in the subpopulation of 4113 participants.

| **Table S3.** C-statistics of models without and with adverse adulthood experiences (AAEs). | | | |
| --- | --- | --- | --- |
|  | **C-statistics (95% CI)^a^** | | **Difference of C-statistics between Model 2 and Model 1 (95% CI)** |
|  | **Unadjusted for AAEs (Model 1)** | **Adjusted for AAEs (Model 2)** |  |
| **Cardiovascular disease** |  |  |  |
| ACEs (1-indicator per increasing)^b^ | 0.50 (0.49-0.51) | 0.50 (0.49-0.52) | 0.00 (-0.01 to 0.01) |
| No. of ACEs indicators |  |  |  |
| 0 | - | - | - |
| ≥1 | 0.50 (0.49-0.51) | 0.50 (0.49-0.52) | 0.00 (-0.01 to 0.01) |
| **Heart disease** |  |  |  |
| ACEs (1-indicator per increasing)^b^ | 0.63 (0.50-0.75) | 0.63 (0.50-0.76) | 0.00 (-0.01 to 0.02) |
| No. of ACEs indicators |  |  |  |
| 0 | - | - |  |
| ≥1 | 0.63 (0.50-0.75) | 0.63 (0.50-0.76) | 0.00 (-0.01 to 0.02) |
| **Stroke** |  |  |  |
| ACEs (1-indicator per increasing)^b^ | 0.68 (0.55-0.79) | 0.68 (0.55-0.80) | 0.00 (-0.01 to 0.02) |
| No. of ACEs indicators |  |  |  |
| 0 | - | - | - |
| ≥1 | 0.68 (0.55-0.79) | 0.68 (0.55-0.80) | 0.00 (-0.01 to 0.02) |

^a^ Models were adjusted for age, sex, residence, marital status, educational level, health behaviors (physical activity, smoking, and drinking), health status variables (body mass index; history of diabetes, hypertension, dyslipidemia, and chronic kidney disease; use of diabetes medications, hypertension medications, and lipid-lowering therapy; and depressive symptoms), and young adulthood social support.

^b^ Continuous variable.

| **Table S4.** Young adulthood social support, sex, and age as effect modifiers for the associations of adverse childhood experiences (ACEs) and adverse adulthood experiences (AAEs) with incident cardiovascular disease (CVD). | | | | | | |
| --- | --- | --- | --- | --- | --- | --- |
|  | **Model 1** | | **Model 2** | | **Model 3** | |
|  | ***β* estimate (95% CI)** | ***P* value** | ***β* estimate (95% CI)** | ***P* value** | ***β* estimate (95% CI)** | ***P* value** |
| **Young adulthood social support** |  |  |  |  |  |  |
| **Cardiovascular disease** |  |  |  |  |  |  |
| ACEs | 0.13 (0.10 to 0.15) | <0.001 | 0.14 (0.12 to 0.17) | <0.001 | 0.12 (0.09 to 0.14) | <0.001 |
| Young adulthood social support | 0.06 (-0.02 to 0.13) | 0.134 | 0.02 (-0.05 to 0.10) | 0.562 | 0.01 (-0.07 to 0.10) | 0.759 |
| ACEs × Young adulthood social support | -0.05 (-0.09 to -0.02) | 0.004 | -0.04 (-0.08 to -0.01) | 0.021 | -0.03 (-0.07 to 0.01) | 0.131 |
| AAEs | 0.21 (0.18 to 0.24) | <0.001 | 0.22 (0.19 to 0.24) | <0.001 | 0.18 (0.15 to 0.21) | <0.001 |
| Young adulthood social support | -0.01 (-0.06 to 0.05) | 0.838 | -0.03 (-0.08 to 0.03) | 0.350 | -0.01 (-0.07 to 0.05) | 0.680 |
| AAEs × Young adulthood social support | -0.04 (-0.08 to -0.01) | 0.026 | -0.04 (-0.08 to 0.002) | 0.062 | -0.03 (-0.07 to 0.01) | 0.095 |
| **Heart disease** |  |  |  |  |  |  |
| ACEs | 0.10 (0.06 to 0.13) | <0.001 | 0.11 (0.07 to 0.15) | <0.001 | 0.07 (0.03 to 0.11) | 0.001 |
| Young adulthood social support | 0.13 (0.03 to 0.24) | 0.014 | 0.10 (0.002 to 0.20) | 0.562 | 0.09 (-0.02 to 0.21) | 0.107 |
| ACEs × Young adulthood social support | -0.07 (-0.12 to -0.02) | 0.008 | -0.06 (-0.12 to -0.001) | 0.021 | -0.04 (-0.10 to 0.01) | 0.119 |
| AAEs | 0.23 (0.19 to 0.27) | <0.001 | 0.24 (0.20 to 0.28) | <0.001 | 0.20 (0.16 to 0.24) | <0.001 |
| Young adulthood social support | 0.06 (-0.03 to 0.14) | 0.181 | 0.03 (-0.05 to 0.12) | 0.407 | 0.08 (-0.003 to 0.17) | 0.061 |
| AAEs × Young adulthood social support | -0.06 (-0.11 to -0.004) | 0.032 | -0.05 (-0.11 to 0.001) | 0.053 | -0.08 (-0.13 to -0.02) | 0.010 |
| **Stroke** |  |  |  |  |  |  |
| ACEs | 0.17 (0.13 to 0.20) | <0.001 | 0.18 (0.15 to 0.22) | <0.001 | 0.16 (0.12 to 0.19) | <0.001 |
| Young adulthood social support | 0.09 (-0.01 to 0.18) | 0.083 | 0.04 (-0.05 to 0.14) | 0.382 | 0.01 (-0.10 to 0.11) | 0.905 |
| ACEs × Young adulthood social support | -0.06 (-0.11 to -0.01) | 0.010 | -0.05 (-0.09 to -0.002) | 0.044 | -0.02 (-0.07 to 0.03) | 0.363 |
| AAEs | 0.18 (0.14 to 0.21) | <0.001 | 0.19 (0.15 to 0.22) | <0.001 | 0.16 (0.12 to 0.19) | <0.001 |
| Young adulthood social support | -0.02 (-0.09 to 0.05) | 0.601 | -0.04 (-0.12 to 0.03) | 0.250 | -0.05 (-0.13 to 0.03) | 0.241 |
| AAEs × Young adulthood social support | -0.01 (-0.06 to 0.04) | 0.742 | -0.0004 (-0.05 to 0.05) | 0.987 | 0.01 (-0.04 to 0.06) | 0.636 |
| **Table S4.** Young adulthood social support, sex, and age as effect modifiers for the associations of adverse childhood experiences (ACEs) and adverse adulthood experiences (AAEs) with incident cardiovascular disease (CVD) (continued). | | | | | | |
| **Biological sex** |  |  |  |  |  |  |
| **Cardiovascular disease** |  |  |  |  |  |  |
| ACEs | 0.08 (0 to 0.16) | 0.042 | 0.06 (-0.02 to 0.14) | 0.110 | 0.03 (-0.05 to 0.11) | 0.529 |
| Biological sex | 0.17 (0.07 to 0.27) | 0.001 | 0.17 (0.05 to 0.29) | 0.005 | 0.10 (-0.02 to 0.22) | 0.106 |
| ACEs × Biological sex | 0.04 (0 to 0.08) | 0.07 | 0.04 (-0.01 to 0.09) | 0.079 | 0.05 (-0.01 to 0.11) | 0.050 |
| AAEs | 0.27 (0.19 to 0.35) | <0.001 | 0.28 (0.20 to 0.36) | <0.001 | 0.21 (0.13 to 0.29) | <0.001 |
| Biological sex | 0.28 (0.20 to 0.36) | <0.001 | 0.29 (0.19 to 0.39) | <0.001 | 0.23 (0.13 to 0.33) | <0.001 |
| AAEs × Biological sex | -0.03 (-0.07 to 0.01) | 0.207 | -0.05 (-0.09 to -0.01) | 0.047 | -0.03 (-0.09 to 0.03) | 0.273 |
| **Heart disease** |  |  |  |  |  |  |
| ACEs | 0.14 (0.10 to 0.18) | <0.001 | -0.01 (-0.12 to 0.11) | 0.890 | -0.08 (-0.20 to 0.04) | 0.218 |
| Biological sex | 0.04 (-0.06 to 0.14) | 0.415 | 0.07 (-0.11 to 0.25) | 0.392 | -0.03 (-0.21 to 0.15) | 0.745 |
| ACEs ×Biological sex | -0.06 (-0.12 to 0) | 0.038 | 0.06 (-0.02 to 0.14) | 0.067 | 0.09 (0.01 to 0.17) | 0.024 |
| AAEs | 0.29 (0.17 to 0.41) | <0.001 | 0.29 (0.17 to 0.41) | <0.001 | 0.24 (0.12 to 0.36) | <0.001 |
| Biological sex | 0.16 (0.04 to 0.28) | 0.002 | 0.24 (0.10 to 0.38) | 0.001 | 0.18 (0.04 to 0.32) | 0.016 |
| AAEs ×Biological sex | -0.03 (-0.11 to 0.05) | 0.404 | -0.05 (-0.13 to 0.03) | 0.171 | -0.04 (-0.12 to 0.04) | 0.287 |
| **Stroke** |  |  |  |  |  |  |
| ACEs | 0.19 (0.15 to 0.23) | <0.001 | 0.11 (0.01 to 0.21) | 0.035 | 0.09 (-0.01 to 0.19) | 0.096 |
| Biological sex | 0.01 (-0.09 to 0.11) | 0.842 | 0.24 (0.09 to 0.39) | 0.002 | 0.21 (0.05 to 0.37) | 0.011 |
| ACEs ×Biological sex | -0.06 (-0.10 to -0.02) | 0.017 | 0.04 (-0.02 to 0.10) | 0.221 | 0.04 (-0.02 to 0.10) | 0.235 |
| AAEs | 0.31 (0.21 to 0.41) | <0.001 | 0.33 (0.23 to 0.43) | <0.001 | 0.26 (0.15 to 0.36) | <0.001 |
| Biological sex | 0.39 (0.29 to 0.49) | <0.001 | 0.38 (0.26 to 0.50) | <0.001 | 0.34 (0.22 to 0.46) | <0.001 |
| AAEs × Biological sex | -0.07 (-0.13 to -0.01) | 0.024 | -0.09 (-0.15 to -0.03) | 0.005 | -0.06 (-0.12 to 0) | 0.064 |
| **Age** |  |  |  |  |  |  |
| **Cardiovascular disease** |  |  |  |  |  |  |
| **Table S4.** Young adulthood social support, sex, and age as effect modifiers for the associations of adverse childhood experiences (ACEs) and adverse adulthood experiences (AAEs) with incident cardiovascular disease (CVD) (continued). | | | | | | |
| ACEs | 0.59 (0.43 to 0.75) | <0.001 | 0.64 (0.48 to 0.80) | <0.001 | 0.68 (0.52 to 0.84) | <0.001 |
| Age | 0.05 (0.04 to 0.06) | <0.001 | 0.05 (0.04 to 0.06) | <0.001 | 0.05 (0.04 to 0.06) | <0.001 |
| ACEs ×Age | -0.01 (-0.01 to -0.01) | <0.001 | -0.01 (-0.01 to -0.01) | <0.001 | -0.01 (-0.01 to -0.01) | <0.001 |
| AAEs | 0.08 (-0.08 to 0.24) | 0.355 | 0.14 (-0.02 to 0.30) | 0.089 | -0.03 (-0.21 to 0.15) | 0.739 |
| Age | 0.03 (0.03 to 0.03) | <0.001 | 0.03 (0.03 to 0.03) | <0.001 | 0.03 (0.03 to 0.03) | <0.001 |
| AAEs × Age | 0.002 (0 to 0.003) | 0.176 | 0.001 (0) | 0.462 | 0.003 (0.001 to 0.01) | 0.025 |
| **Heart disease** |  |  |  |  |  |  |
| ACEs | 0.57 (0.35 to 0.79) | <0.001 | 0.63 (0.41 to 0.85) | <0.001 | 0.68 (0.04 to 0.93) | <0.001 |
| Age | 0.06 (0.05 to 0.07) | <0.001 | 0.06 (0.05 to 0.07) | <0.001 | 0.05 (0.04 to 0.06) | <0.001 |
| ACEs × Age | -0.008 (-0.01 to 0) | <0.001 | -0.01 (-0.01 to -0.01) | <0.001 | -0.01 (-0.01 to -0.01) | <0.001 |
| AAEs | 0.47 (0.24 to 0.71) | <0.001 | 0.51 (0.27 to 0.75) | <0.001 | 0.27 (0.02 to 0.52) | 0.044 |
| Age | 0.05 (0.04 to 0.05) | <0.001 | 0.05 (0.04 to 0.06) | <0.001 | 0.04 (0.03 to 0.05) | <0.001 |
| AAEs × Age | -0.004 (-0.008 to -0.001) | 0.026 | -0.01 (-0.01 to -0.01) | 0.012 | -0.002 (-0.01 to 0.002) | 0.479 |
| **Stroke** |  |  |  |  |  |  |
| ACEs | 0.62 (0.43 to 0.81) | <0.001 | 0.68 (0.48 to 0.88) | <0.001 | 0.74 (0.52 to 0.96) | <0.001 |
| Age | 0.04 (0.04 to 0.05) | <0.001 | 0.05 (0.04 to 0.06) | <0.001 | 0.04 (0.03 to 0.05) | <0.001 |
| ACEs × Age | -0.01 (-0.01 to 0) | <0.001 | -0.01 (-0.01 to -0.01) | <0.001 | -0.01 (-0.01 to -0.01) | <0.001 |
| AAEs | -0.10 (-0.31 to 0.11) | 0.343 | -0.03 (-0.25 to 0.19) | 0.758 | -0.14 (-0.38 to 0.09) | 0.214 |
| Age | 0.03 (0.02 to 0.04) | <0.001 | 0.03 (0.02 to 0.04) | <0.001 | 0.02 (0.01 to 0.03) | <0.001 |
| AAEs × Age | 0.01 (0.001 to 0.01) | 0.012 | 0.004 (0 to 0.01) | 0.042 | 0.01 (0.001 to 0.01) | 0.008 |

Abbreviation: ACEs, adverse childhood experiences; AAEs, adverse adulthood experiences; 95% CI, 95% confidence interval.

Model 1 was adjusted for age and sex.

Model 2 for assessing the interaction term (ACEs/AAEs × young adulthood social support) was adjusted for age, sex, residence, educational level, marital status, physical activity, smoking status, and drinking status. Model 2 for assessing the interaction term (ACEs/AAEs × sex) was adjusted for age, residence, educational level, marital status, physical activity, smoking status, and drinking status. Model 2 for assessing the interaction term (ACEs/AAEs × age) was adjusted for sex, residence, educational level, marital status, physical activity, smoking status, and drinking status.

Model 3 was adjusted as Model 2 plus body mass index; history of diabetes, hypertension, dyslipidemia, and chronic kidney disease; use of diabetes medications, hypertension medications, and lipid-lowering therapy; and depressive symptoms score.

**Table S5.** Hazard ratios for associations of adverse childhood experiences (ACEs), adverse adulthood experience (AAEs), and young adulthood social support with incident cardiovascular disease (CVD): subgroup analyses.

|  | **HR (95% CI)^a^** | | | |
| --- | --- | --- | --- | --- |
|  | **Males** | **Females** | **Adults aged< 60 years** | **Adults aged ≥60 years** |
| **Cardiovascular disease** |  |  |  |  |
| ACEs indicators^b^ (1-unit per increasing) | 1.09 (1.05-1.13) | 1.13 (1.09-1.17) | 1.16 (1.12-1.20) | 1.03 (0.99-1.07) |
| No. of ACEs indicators |  |  |  |  |
| 0 | 1.00 (Reference) | 1.00 (Reference) | 1.00 (Reference) | 1.00 (Reference) |
| 1 | 1.54 (1.31-1.80) | 1.11 (0.98-1.26) | 1.43 (1.25-1.62) | 0.90 (0.77-1.06) |
| 2 | 1.35 (1.14-1.60) | 1.42 (1.25-1.63) | 1.43 (1.24-1.65) | 1.05 (0.89-1.23) |
| 3 | 1.83 (1.53-2.19) | 1.38 (1.19-1.60) | 1.82 (1.57-2.11) | 1.03 (0.87-1.23) |
| ≥4 | 1.39 (1.11-1.75) | 1.73 (1.46-2.06) | 1.85 (1.54-2.23) | 1.00 (0.82-1.23) |
| AAEs indicators^b^ (1-unit per increasing) | 1.21 (1.17-1.26) | 1.17 (1.13-1.21) | 1.12 (1.08-1.16) | 1.25 (1.20-1.30) |
| No. of AAEs indicators |  |  |  |  |
| 0 | 1.00 (Reference) | 1.00 (Reference) | 1.00 (Reference) | 1.00 (Reference) |
| 1 | 1.13 (1.00-1.28) | 1.10 (0.99-1.22) | 0.98 (0.88-1.10) | 1.22 (1.09-1.37) |
| 2 | 1.29 (1.11-1.50) | 1.56 (1.37-1.77) | 1.23 (1.06-1.43) | 1.61 (1.41-1.84) |
| 3 | 1.77 (1.52-2.07) | 1.60 (1.38-1.87) | 1.51 (1.30-1.76) | 1.78 (1.52-2.08) |
| ≥4 | 2.47 (2.00-3.05) | 1.57 (1.22-2.03) | 1.46 (1.14-1.87) | 2.54 (2.05-3.15) |
| Young adulthood social support^b^ (1-unit per increasing) | 1.01 (0.95-1.08) | 0.90 (0.84-0.97) | 1.00 (0.94-1.06) | 1.00 (1.00-1.01) |
| **Heart disease** |  |  |  |  |
| ACEs indicators^b^ (1-unit per increasing) | 1.01 (0.96-1.07) | 1.09 (1.04-1.15) | 1.10 (1.05-1.16) | 0.97 (0.91-1.02) |
| No. of ACEs indicators |  |  |  |  |
| 0 | 1.00 (Reference) | 1.00 (Reference) | 1.00 (Reference) | 1.00 (Reference) |
| 1 | 1.49 (1.20-1.86) | 1.17 (0.96-1.41) | 1.28 (1.06-1.54) | 1.14 (0.90-1.44) |
| 2 | 1.02 (0.80-1.30) | 1.44 (1.17-1.76) | 1.28 (1.04-1.58) | 0.99 (0.77-1.26) |
|  | | | | |
| **Table S5.** Hazard ratios for associations of adverse childhood experiences (ACEs), adverse adulthood experience (AAEs), and young adulthood social support with incident cardiovascular disease (CVD): subgroup analyses (continued). | | | | |
| 3 | 1.35 (1.05-1.75) | 1.44 (1.15-1.80) | 1.62 (1.30-2.02) | 0.97 (0.74-1.27) |
| ≥4 | 1.29 (0.94-1.75) | 1.47 (1.12-1.92) | 1.41 (1.06-1.86) | 1.00 (0.74-1.37) |
| AAEs indicators^b^ (1-unit per increasing) | 1.22 (1.16-1.29) | 1.17 (1.11-1.24) | 1.21 (1.14-1.27) | 1.15 (1.09-1.22) |
| No. of AAEs indicators |  |  |  |  |
| 0 | 1.00 (Reference) | 1.00 (Reference) | 1.00 (Reference) | 1.00 (Reference) |
| 1 | 1.19 (1.00-1.42) | 1.20 (1.03-1.41) | 1.13 (0.96-1.34) | 1.18 (1.00-1.39) |
| 2 | 1.56 (1.27-1.92) | 1.70 (1.40-2.07) | 1.55 (1.26-1.90) | 1.56 (1.28-1.89) |
| 3 | 1.39 (1.09-1.77) | 1.68 (1.34-2.10) | 1.77 (1.42-2.20) | 1.18 (0.91-1.53) |
| ≥4 | 2.99 (2.26-3.95) | 1.09 (0.67-1.77_ | 2.02 (1.45-2.80) | 1.90 (1.34-2.70) |
| Young adulthood social support^b^ (1-unit per increasing) | 1.04 (1.03-1.05) | 1.03 (1.03-1.04) | 1.01 (0.92-1.10) | 1.06 (0.96-1.18) |
| **Stroke** |  |  |  |  |
| ACEs indicators^b^ (1-unit per increasing) | 1.15 (1.09-1.21) | 1.18 (1.13-1.23) | 1.21 (1.16-1.26) | 1.09 (1.04-1.15) |
| No. of ACEs indicators |  |  |  |  |
| 0 | 1.00 (Reference) | 1.00 (Reference) | 1.00 (Reference) | 1.00 (Reference) |
| 1 | 1.73 (1.39-2.17) | 1.10 (0.93-1.29) | 1.63 (1.38-1.93) | 0.77 (0.63-0.94) |
| 2 | 1.77 (1.40-2.24) | 1.54 (1.31-1.83) | 1.64 (1.36-1.97) | 1.15 (0.94-1.41) |
| 3 | 2.53 (1.99-3.21) | 1.39 (1.15-1.68) | 2.08 (1.71-2.52) | 1.10 (0.89-1.37) |
| ≥4 | 1.50 (1.09-2.05) | 2.13 (1.73-2.62) | 2.42 (1.92-3.04) | 1.05 (0.81-1.36) |
| AAEs indicators^b^ (1-unit per increasing) | 1.23 (1.17-1.29) | 1.14 (1.09-1.19) | 1.08 (1.03-1.14) | 1.27 (1.21-1.33) |
| No. of AAEs indicators |  |  |  |  |
| 0 | 1.00 (Reference) | 1.00 (Reference) | 1.00 (Reference) | 1.00 (Reference) |
| 1 | 1.12 (0.95-1.32) | 1.04 (0.91-1.18) | 0.96 (0.83-1.11) | 1.18 (1.02-1.36) |
| 2 | 1.09 (0.89-1.35) | 1.38 (1.17-1.63) | 1.00 (0.81-1.22) | 1.50 (1.26-1.78) |
| 3 | 2.10 (1.73-2.54) | 1.34 (1.09-1.65) | 1.38 (1.13-1.69) | 1.98 (1.63-2.41) |
| ≥4 | 2.57 (1.96-3.38) | 1.88 (1.41-2.51) | 1.48 (1.09-2.01) | 2.86 (2.20-3.71) |

Abbreviation: ACEs, adverse childhood experiences; AAEs, adverse adulthood experiences; HR, hazard ratio; 95% CI, 95% confidence interval.

^a^ Models were adjusted for age, sex, residence, marital status, educational level, health behaviors (physical activity, smoking, and drinking), health status variables (body mass index; history of diabetes, hypertension, dyslipidemia, and chronic kidney disease; use of diabetes medications, hypertension medications, and lipid-lowering therapy; and depressive symptoms), and young adulthood social support.

^b^ Continuous variable.

| **Table S6.** Associations of adverse childhood experiences (ACEs) with incident cardiovascular disease (CVD) and mediation proportion of the associations attributed to adverse adulthood experience (AAEs): subgroup analyses. | | | |
| --- | --- | --- | --- |
| **Subgroups** | **HR (95% CI)^a^** | | **Mediation proportion (%) (95% CI)** |
|  | **Unadjusted for AAEs** | **Adjusted for AAEs** |  |
| **Cardiovascular disease** |  |  |  |
| **Males** |  |  |  |
| ACEs (1 indicator per increasing)^b^ | 1.09 (1.05-1.13) | 1.07 (1.03-1.12) | 19.1 (4.6-53.4) |
| No. of ACEs indicators |  |  |  |
| 0 | 1.00 (Reference) | 1.00 (Reference) | - |
| ≥1 | 1.52 (1.31-1.76) | 1.49 (1.29-1.74) | 3.9 (0.5-23.5) |
| **Females** |  |  |  |
| ACEs (1 indicator per increasing)^b^ | 1.13 (1.09-1.17) | 1.11 (1.07-1.15) | 15.2 (5.7-35.0) |
| No. of ACEs indicators |  |  |  |
| 0 | 1.00 (Reference) | 1.00 (Reference) | - |
| ≥1 | 1.29 (1.15-1.44) | 1.26 (1.13-1.42) | 6.5 (1.3-27.2) |
| **Adults aged< 60 years** |  |  |  |
| ACEs (1 indicator per increasing)^b^ | 1.16 (1.12-1.20) | 1.15 (1.11-1.19) | 5.8 (1.6-19.0) |
| No. of ACEs indicators |  |  |  |
| 0 | 1.00 (Reference) | 1.00 (Reference) | - |
| ≥1 | 1.46 (1.30-1.63) | 1.44 (1.29-1.62) | 2.6 (0.6-11.5) |
| **Adults aged≥ 60 years** |  |  |  |
| ACEs (1 indicator per increasing)^b^ | 1.03 (0.99-1.07) | 1.00 (0.95-1.03) | NI |
| No. of ACEs indicators |  |  |  |
| 0 | 1.00 (Reference) | 1.00 (Reference) | - |
| ≥1 | 1.03 (0.89-1.21) | 1.01 (0.88-1.20) | NI |
| **Heart disease** |  |  |  |
| **Males** |  |  |  |
| ACEs (1 indicator per increasing)^b^ | 1.01 (0.96-1.07) | 1.00 (0.94-1.06) | NI |
| No. of ACEs indicators |  |  |  |
| 0 | 1.00 (Reference) | 1.00 (Reference) | - |
| ≥1 | 1.31 (1.07-1.61) | 1.29 (1.05-1.58) | 6.9 (0.5-54.2) |
| **Females** |  |  |  |
| ACEs (1 indicator per increasing)^b^ | 1.09 (1.04-1.15) | 1.07 (1.02-1.13) | 23.3 (3.6-71.4) |
| No. of ACEs indicators |  |  |  |
| **Table S6.** Associations of adverse childhood experiences (ACEs) with incident cardiovascular disease (CVD) and mediation proportion of the associations attributed to adverse adulthood experience (AAEs): subgroup analyses (continued). | | | |
| 0 | 1.00 (Reference) | 1.00 (Reference) | - |
| ≥1 | 1.31 (1.09-1.56) | 1.29 (1.08-1.53) | 5.8 (0.6-38.0) |
| **Adults aged< 60 years** |  |  |  |
| ACEs (1 indicator per increasing)^b^ | 1.10 (1.05-1.16) | 1.10 (1.05-1.16) | 12.3 (2.5-43.5) |
| No. of ACEs indicators |  |  |  |
| 0 | 1.00 (Reference) | 1.00 (Reference) | - |
| ≥1 | 1.29 (1.09-1.53) | 1.27 (1.07-1.50) | 6.6 (0.8-39.2) |
| **Adults aged≥ 60 years** |  |  |  |
| ACEs (1 indicator per increasing)^b^ | 0.97 (0.91-1.02) | 0.92 (0.87-0.98) | NI |
| No. of ACEs indicators |  |  |  |
| 0 | 1.00 (Reference) | 1.00 (Reference) | - |
| ≥1 | 1.12 (0.89-1.42) | 1.10 (0.87-1.39) | 14.0 (0-98.7) |
| **Stroke** |  |  |  |
| **Males** |  |  |  |
| ACEs (1 indicator per increasing)^b^ | 1.15 (1.09-1.21) | 1.13 (1.07-1.19) | 13.2 (3.7-37.6) |
| No. of ACEs indicators |  |  |  |
| 0 | 1.00 (Reference) | 1.00 (Reference) | - |
| ≥1 | 1.85 (1.50-2.29) | 1.82 (1.48-2.25) | 2.7 (0.3-18.2) |
| **Females** |  |  |  |
| ACEs (1 indicator per increasing)^b^ | 1.18 (1.13-1.23) | 1.17 (1.12-1.22) | 8.0 (2.0-27.4) |
| No. of ACEs indicators |  |  |  |
| 0 | 1.00 (Reference) | 1.00 (Reference) | - |
| ≥1 | 1.34 (1.16-1.55) | 1.32 (1.14-1.53) | 4.6 (0.7-24.4) |
| **Adults aged< 60 years** |  |  |  |
| ACEs (1 indicator per increasing)^b^ | 1.21 (1.16-1.26) | 1.20 (1.15-1.25) | 2.7 (0.2-30.1) |
| No. of ACEs indicators |  |  |  |
| 0 | 1.00 (Reference) | 1.00 (Reference) | - |
| ≥1 | 1.69 (1.45-1.97) | 1.68 (1.44-1.96) | 1.3 (0.1-10.5) |
| **Adults aged≥ 60 years** |  |  |  |
| ACEs (1 indicator per increasing)^b^ | 1.09 (1.04-1.15) | 1.05 (1.00-1.11) | 37.7 (4.9-87.7) |
| No. of ACEs indicators |  |  |  |
| 0 | 1.00 (Reference) | 1.00 (Reference) | - |
| ≥1 | 1.01 (0.83-1.22) | 0.98 (0.80-1.19) | NI |

Abbreviation: ACEs, adverse childhood experiences; AAEs, adverse adulthood experiences; HR, hazard ratio; 95% CI, 95% confidence interval; NI, not intermediate.

^a^ Models were adjusted for age, sex, residence, marital status, educational level, health behaviors (physical activity, smoking, and drinking), health status variables (BMI; history of diabetes, hypertension, dyslipidemia, and chronic kidney disease; use of diabetes medications, hypertension medications, and lipid-lowering therapy; and depressive symptoms), and young adulthood social support.

^b^ Continuous variable.

| **Table S7.** Associations of adverse childhood experience (ACEs) and adverse adulthood experiences (AAEs) with incident cardiovascular disease (CVD) in the subpopulation of 4113 participants with metabolic biomarkers measurements. | | | | | | |
| --- | --- | --- | --- | --- | --- | --- |
|  | **HR (95% CI)** | | | | | |
|  | **Model 4** | **Model 5** | **Model 6** | **Model 7** | **Model 8** | **Model 9** |
| ACEs (1 indicator per increasing)^a^ | 1.15 (1.06-1.25) | 1.14 (1.05-1.24) | 1.14 (1.05-1.24) | 1.15 (1.05-1.25) | 1.15 (1.05-1.25) | 1.14 (1.05-1.24) |
| No. of ACEs indicators |  |  |  |  |  |  |
| 0 | 1.00 (Reference) | 1.00 (Reference) | 1.00 (Reference) | 1.00 (Reference) | 1.00 (Reference) | 1.00 (Reference) |
| 1 | 1.51 (1.07-2.14) | 1.50 (1.06-2.12) | 1.49 (1.05-2.11) | 1.52 (1.07-2.14) | 1.51 (1.07-2.13) | 1.49 (1.05-2.10) |
| 2 | 1.78 (1.25-2.55) | 1.75 (1.22-2.50) | 1.79 (1.25-2.56) | 1.78 (1.24-2.55) | 1.77 (1.24-2.53) | 1.76 (1.23-2.52) |
| 3 | 1.65 (1.11-2.45) | 1.62 (1.09-2.41) | 1.62 (1.09-2.41) | 1.63 (1.10-2.43) | 1.62 (1.09-2.41) | 1.61 (1.08-2.39) |
| ≥4 | 2.12 (1.35-3.33) | 2.02 (1.28-3.19) | 1.99 (1.26-3.15) | 2.07 (1.31-3.26) | 2.07 (1.31-3.27) | 2.02 (1.28-3.20) |
| AAEs (1 indicator per increasing)^a^ | 1.19 (1.09-1.30) | 1.19 (1.09-1.30) | 1.19 (1.09-1.30) | 1.19 (1.09-1.30) | 1.19 (1.09-1.30) | 1.19 (1.09-1.30) |
| No. of AAEs indicators |  |  |  |  |  |  |
| 0 | 1.00 (Reference) | 1.00 (Reference) | 1.00 (Reference) | 1.00 (Reference) | 1.00 (Reference) | 1.00 (Reference) |
| 1 | 1.10 (0.85-1.42) | 1.10 (0.85-1.42) | 1.10 (0.85-1.42) | 1.09 (0.85-1.41) | 1.09 (0.85-1.41) | 1.09 (0.85-1.42) |
| 2 | 1.39 (1.00-1.94) | 1.42 (1.01-1.99) | 1.39 (1.00-1.96) | 1.41 (1.01-1.98) | 1.41 (1.01-1.98) | 1.41 (1.00-1.98) |
| 3 | 1.81 (1.25-2.63) | 1.82 (1.26-2.64) | 1.79 (1.23-2.60) | 1.81 (1.25-2.63) | 1.83 (1.27-2.66) | 1.80 (1.24-2.62) |
| ≥4 | 1.92 (1.11-3.33) | 1.92 (1.11-3.33) | 1.94 (1.12-3.36) | 1.92 (1.11-3.23) | 1.94 (1.12-3.35) | 1.94 (1.12-3.36) |

Abbreviation: ACEs, adverse childhood experiences; AAEs, adverse adulthood experiences; No., number; HR, hazard ratio; 95% CI, 95% confidence interval.

^a^ Continuous variable.

Model 4 was adjusted for age, sex, residence, marital status, educational level, health behaviors (physical activity, smoking, and drinking), health status variables (body mass index; history of diabetes, hypertension, dyslipidemia, and chronic kidney disease; use of diabetes medications, hypertension medications, and lipid-lowering therapy; and depressive symptoms), and young adulthood social support.

Model 5 was adjusted as Model 4 plus fasting plasma glucose and glycosylated hemoglobin.

Model 6 was adjusted as Model 4 plus total cholesterol, triglyceride, high-density lipoprotein, and low-density lipoprotein.

Model 7 was adjusted as Model 4 plus high-sensitivity C-reactive protein.

Model 8 was adjusted as Model 4 plus the estimated glomerular filtration rate.

Model 9 was adjusted as Model 4 plus all biomarkers.

**Table S8.** Hazard ratios for associations of adverse childhood experiences (ACEs), adverse adulthood experience (AAEs), and young adulthood social support with incident cardiovascular disease (CVD): using the complete dataset.

|  | **HR (95% CI)** | | | |
| --- | --- | --- | --- | --- |
|  | **Model 1** | **Model 2** | **Model 3** | **Model 4** |
| **Cardiovascular disease** |  |  |  |  |
| ACEs indicators^a^ (1-unit per increasing) | 1.12 (1.05-1.20) | 1.15 (1.08-1.23) | 1.13 (1.05-1.22) | 1.13 (1.05-1.22) |
| No. of ACEs indicators |  |  |  |  |
| 0 | 1.00 (Reference) | 1.00 (Reference) | 1.00 (Reference) | 1.00 (Reference) |
| 1 | 1.25 (0.98-1.61) | 1.29 (1.00-1.66) | 1.32 (0.99-1.76) | 1.32 (0.99-1.76) |
| 2 | 1.38 (1.06-1.80) | 1.46 (1.12-1.91) | 1.51 (1.12-2.05) | 1.52 (1.12-2.06) |
| 3 | 1.51 (1.13-2.01) | 1.63 (1.22-2.18) | 1.51 (1.08-2.10) | 1.52 (1.09-2.12) |
| ≥4 | 1.69 (1.20-2.37) | 1.86 (1.32-2.62) | 1.80 (1.22-2.66) | 1.83 (1.24-2.70) |
| AAEs indicators^a^ (1-unit per increasing) | 1.18 (1.10-1.26) | 1.19 (1.11-1.28) | 1.17 (1.09-1.27) | 1.18 (1.09-1.27) |
| No. of AAEs indicators |  |  |  |  |
| 0 | 1.00 (Reference) | 1.00 (Reference) | 1.00 (Reference) | 1.00 (Reference) |
| 1 | 1.11 (0.90-1.35) | 1.15 (0.94-1.41) | 1.08 (0.86-1.35) | 1.08 (0.86-1.35) |
| 2 | 1.31 (1.01-1.71) | 1.35 (1.04-1.76) | 1.38 (1.03-1.85) | 1.38 (1.03-1.85) |
| 3 | 1.76 (1.32-2.35) | 1.82 (1.36-2.43) | 1.71 (1.24-2.37) | 1.74 (1.26-2.40) |
| ≥4 | 1.87 (1.22-2.88) | 1.94 (1.26-2.98) | 1.83 (1.13-2.95) | 1.83 (1.13-2.96) |
| Young adulthood social support^a^ (1-unit per increasing) | 0.94 (0.83-1.07) | 0.93 (0.82-1.06) | 0.93 (0.81-1.08) | NA |
| **Heart disease** |  |  |  |  |
| ACEs indicators^a^ (1-unit per increasing) | 1.04 (0.92-1.18) | 1.06 (0.93-1.19) | 1.08 (0.94-1.24) | 1.08 (0.94-1.24) |
| No. of ACEs indicators |  |  |  |  |
| 0 | 1.00 (Reference) | 1.00 (Reference) | 1.00 (Reference) | 1.00 (Reference) |
| **Table S8.** Hazard ratios for associations of adverse childhood experiences (ACEs), adverse adulthood experience (AAEs), and young adulthood social support with incident cardiovascular disease (CVD): using the complete dataset (continued). | | | | |
| 1 | 1.29 (0.82-2.03) | 1.33 (0.85-2.09) | 1.59 (0.93-2.74) | 1.59 (0.92-2.74) |
| 2 | 1.22 (0.75-1.98) | 1.26 (0.78-2.05) | 1.61 (0.91-2.85) | 1.61 (0.91-2.85) |
| 3 | 1.27 (0.75-2.17) | 1.35 (0.79-2.31) | 1.61 (0.86-3.02) | 1.61 (0.86-3.02) |
| ≥4 | 1.34 (0.70-2.57) | 1.41 (0.73-2.73) | 1.64 (0.77-3.50) | 1.64 (0.77-3.51) |
| AAEs indicators^a^ (1-unit per increasing) | 1.12 (0.98-1.28) | 1.12 (0.98-1.28) | 1.17 (1.01-1.35) | 1.17 (1.01-1.35) |
| No. of AAEs indicators |  |  |  |  |
| 0 | 1.00 (Reference) | 1.00 (Reference) | 1.00 (Reference) | 1.00 (Reference) |
| 1 | 1.21 (0.85-1.72) | 1.23 (0.86-1.76) | 1.17 (0.79-1.73) | 1.17 (0.79-1.73) |
| 2 | 1.04 (0.62-1.74) | 1.03 (0.61-1.74) | 1.37 (0.81-2.34) | 1.38 (0.81-2.35) |
| 3 | 1.31 (0.72-2.38) | 1.31 (0.72-2.39) | 1.41 (0.73-2.73) | 1.41 (0.73-2.73) |
| ≥4 | 2.14 (0.99-4.60) | 2.14 (1.00-4.62) | 2.13 (0.92-4.93) | 2.13 (0.92-4.93) |
| Young adulthood social support^a^ (1-unit per increasing) | 0.97 (0.77-1.23) | 0.95 (0.75-1.21) | 1.01 (0.79-1.31) | NA |
| **Stroke** |  |  |  |  |
| ACEs indicators^a^ (1-unit per increasing) | 1.21 (1.11-1.32) | 1.24 (1.13-1.35) | 1.20 (1.09-1.33) | 1.20 (1.09-1.33) |
| No. of ACEs indicators |  |  |  |  |
| 0 | 1.00 (Reference) | 1.00 (Reference) | 1.00 (Reference) | 1.00 (Reference) |
| 1 | 1.58 (1.08-2.33) | 1.62 (1.10-2.37) | 1.64 (1.06-2.53) | 1.64 (1.06-2.53) |
| 2 | 2.02 (1.36-2.99) | 2.12 (1.43-3.14) | 2.11 (1.35-3.29) | 2.10 (1.34-3.27) |
| 3 | 2.00 (1.30-3.07) | 2.14 (1.38-3.30) | 1.88 (1.15-3.06) | 1.87 (1.15-3.05) |
| ≥4 | 2.52 (1.55-4.09) | 2.76 (1.69-4.50) | 2.60 (1.51-4.84) | 2.58 (1.49-4.45) |
| AAEs indicators^a^ (1-unit per increasing) | 1.20 (1.09-1.33) | 1.22 (1.11-1.34) | 1.18 (1.06-1.32) | 1.18 (1.06-1.31) |
| No. of AAEs indicators |  |  |  |  |
| **Table S8.** Hazard ratios for associations of adverse childhood experiences (ACEs), adverse adulthood experience (AAEs), and young adulthood social support with incident cardiovascular disease (CVD): using the complete dataset (continued). | | | | |
| 0 | 1.00 (Reference) | 1.00 (Reference) | 1.00 (Reference) | 1.00 (Reference) |
| 1 | 1.13 (0.85-1.50) | 1.19 (0.89-1.58) | 1.04 (0.76-1.42) | 1.03 (0.75-1.42) |
| 2 | 1.42 (0.99-2.04) | 1.48 (1.03-2.13) | 1.33 (0.88-2.01) | 1.33 (0.88-2.01) |
| 3 | 1.88 (1.26-2.80) | 1.92 (1.29-2.86) | 1.84 (1.19-2.85) | 1.82 (1.18-2.82) |
| ≥4 | 2.05 (1.11-3.77) | 2.12 (1.15-3.90) | 1.91 (1.00-3.67) | 1.89 (1.00-3.63) |
| Young adulthood social support^a^ (1-unit per increasing) | 1.06 (0.89-1.26) | 1.05 (0.88-1.24) | 1.08 (0.89-1.30) | NA |

Abbreviation: ACEs, adverse childhood experiences; AAEs, adverse adulthood experiences; No., number; HR, hazard ratio; 95% CI, 95% confidence interval; NA, not available or not applicable.

^a^ Continuous variable.

Model 1 was adjusted for age and sex.

Model 2 was adjusted for age, sex, residence, educational level, marital status, physical activity, smoking status, and drinking status.

Model 3 was adjusted as Model 2 plus body mass index; history of diabetes, hypertension, dyslipidemia, and chronic kidney disease; use of diabetes medications, hypertension medications, and lipid-lowering therapy; and depressive symptoms score.

Model 4 was adjusted as Model 3 plus young adulthood social support.

| **Table S9.** Associations of adverse childhood experiences (ACEs) with incident cardiovascular disease (CVD) and mediation proportion of the associations attributed to adverse adulthood experiences (AAEs): using the complete dataset. | | | |
| --- | --- | --- | --- |
|  | **HR (95% CI)^a^** | | **Mediation proportion (%) (95% CI)** |
|  | **Unadjusted for AAEs** | **Adjusted for AAEs** |  |
| **Cardiovascular disease** |  |  |  |
| ACEs indicators^b^ (1-unit per increasing) | 1.13 (1.05-1.22) | 1.12 (1.04-1.20) | 12.5 (5.2-27.1) |
| No. of ACEs indicators |  |  |  |
| 0 | 1.00 (Reference) | 1.00 (Reference) | - |
| ≥1 | 1.44 (1.11-1.86) | 1.42 (1.10-1.84) | 2.8 (0.5-14.3) |
| **Heart Disease** |  |  |  |
| ACEs indicators^b^ (1-unit per increasing) | 1.08 (0.94-1.24) | 1.07 (0.93-1.22) | 17.1 (2.3-64.0) |
| No. of ACEs indicators |  |  |  |
| 0 | 1.00 (Reference) | 1.00 (Reference) | - |
| ≥1 | 1.60 (0.96-2.67) | 1.58 (0.95-2.65) | 2.2 (0.2-18.7) |
| **Stroke** |  |  |  |
| ACEs indicators^b^ (1-unit per increasing) | 1.20 (1.09-1.33) | 1.18 (1.07-1.31) | 7.8 (2.7-20.5) |
| No. of ACEs indicators |  |  |  |
| 0 | 1.00 (Reference) | 1.00 (Reference) | - |
| ≥1 | 1.88 (1.25-2.82) | 1.86 (1.24-2.80) | 1.3 (0.1-14.1) |

Abbreviation: ACEs, adverse childhood experiences; AAEs, adverse adulthood experiences; HR, hazard ratio; 95% CI, 95% confidence interval.

^a^ Models were adjusted for age, sex, residence, marital status, educational level, health behaviors (physical activity, smoking, and drinking), health status variables (BMI; history of diabetes, hypertension, dyslipidemia, and chronic kidney disease; use of diabetes medications, hypertension medications, and lipid-lowering therapy; and depressive symptoms), and adulthood social support.

^b^ Continuous variable.

| **Table S10.** Hazard ratios for associations of each indicator of adverse childhood experiences (ACEs) and adverse adulthood experiences (AAEs) with incident cardiovascular disease (CVD). | | | | |
| --- | --- | --- | --- | --- |
| **Indicators** | **HR (95% CI)** | | | |
|  | **Model 1** | **Model 2** | **Model 3** | **Model 4** |
| No indicators | 1.00 (Reference) | 1.00 (Reference) | 1.00 (Reference) | 1.00 (Reference) |
| Adverse childhood experience (yes) |  |  |  |  |
| Physical neglect | 1.14 (1.07-1.22) | 1.19 (1.11-1.27) | 1.16 (1.08-1.24) | 1.15 (1.07-1.24) |
| Emotional neglect | 0.90 (0.75-1.07) | 0.88 (0.74-1.06) | 0.96 (0.78-1.18) | 0.96 (0.78-1.18) |
| Domestic violence | 1.34 (1.22-1.47) | 1.35 (1.23-1.48) | 1.34 (1.22-1.48) | 1.35 (1.22-1.48) |
| Criminal behavior in the household | 1.20 (0.80-1.79) | 1.24 (0.83-1.85) | 1.27 (0.80-2.02) | 1.26 (0.79-2.01) |
| Parental separation or divorce | 0.41 (0.06-2.86) | 0.38 (0.05-2.73) | 0.48 (0.07-3.45) | 0.48 (0.07-3.45) |
| Parental death | 1.03 (0.96-1.01) | 1.06 (0.99-1.14) | 1.10 (1.02-1.18) | 1.10 (1.03-1.19) |
| Physical abuse | 1.24 (1.16-1.33) | 1.27 (1.18-1.36) | 1.19 (1.11-1.28) | 1.19 (1.11-1.28) |
| Household mental illness | 1.16 (1.09-1.24) | 1.21 (1.13-1.29) | 1.13 (1.05-1.21) | 1.13 (1.06-1.22) |
| Exposure to natural disasters | 1.51 (1.38-1.65) | 1.53 (1.39-1.68) | 1.29 (1.17-1.42) | 1.29 (1.17-1.42) |
| Household substance use | 1.05 (0.95-1.17) | 1.06 (0.96-1.18) | 1.05 (0.94-1.17) | 1.05 (0.94-1.17) |
| Adverse adulthood experience (yes) |  |  |  |  |
| Death of the child | 1.01 (0.92-1.09) | 1.05 (0.97-1.15) | 1.03 (0.94-1.13) | 1.03 (0.94-1.12) |
| Experiencing lifetime discrimination | 1.09 (0.99-1.20) | 1.13 (1.03-1.24) | 1.08 (0.98-1.18) | 1.08 (0.98-1.19) |
| Ever being confined to bed | 1.81 (1.69-1.93) | 1.83 (1.71-1.96) | 1.70 (1.58-1.82) | 1.70 (1.58-1.83) |
| Ever being hospitalized for a month or more | 1.76 (1.63-1.90) | 1.73 (1.60-1.86) | 1.53 (1.41-1.66) | 1.53 (1.41-1.66) |
| Ever leaving a job due to health conditions | 1.48 (1.38-1.58) | 1.51 (1.41-1.61) | 1.44 (1.33-1.54) | 1.44 (1.34-1.55) |

Abbreviation: ACEs, adverse childhood experiences; AAEs, adverse adulthood experiences.

Model 1 was adjusted for age and sex.

Model 2 was adjusted for age, sex, residence, educational level, marital status, physical activity, smoking status, and drinking status.

Model 3 was adjusted as Model 2 plus body mass index; history of diabetes, hypertension, dyslipidemia, and chronic kidney disease; use of diabetes medications, hypertension medications, and lipid-lowering therapy; and depressive symptoms score.

Model 4 was adjusted as Model 3 plus young adulthood social support.

| **Table S11.** Associations of each indicator of adverse childhood experiences (ACEs) with incident cardiovascular disease (CVD) and mediation proportion of the associations attributed to adverse adulthood experiences (AAEs). | | | |
| --- | --- | --- | --- |
| **Indicators of adverse childhood experience** | **HR (95% CI)^a^** | | **Mediation proportion (%) (95% CI)** |
|  | **Unadjusted for AAEs** | **Adjusted for AAEs** |  |
| No indicators | 1.00 (Reference) | 1.00 (Reference) |  |
| Physical neglect | 1.15 (1.07-1.24) | 1.13 (1.06-1.22) | 10.2 (2.0-38.5) |
| Emotional neglect | 0.96 (0.78-1.18) | 0.97 (0.79-1.19) | NI |
| Domestic violence | 1.35 (1.22-1.48) | 1.31 (1.19-1.44) | 9.1 (2.8-26.1) |
| Criminal behavior in the household | 1.26 (0.79-2.01) | 1.21 (0.76-1.92) | NI |
| Parental separation or divorce | 0.48 (0.07-3.45) | 0.48 (0.07-3.43) | NI |
| Parental death | 1.10 (1.03-1.19) | 1.09 (1.01-1.17) | 6.7 (0.9-36.6) |
| Physical abuse | 1.19 (1.11-1.28) | 1.15 (1.07-1.24) | 17.8 (5.1-46.4) |
| Household mental illness | 1.13 (1.06-1.22) | 1.08 (1.01-1.16) | 39.7 (6.4-86.4) |
| Exposure to natural disasters | 1.29 (1.17-1.42) | 1.26 (1.15-1.39) | 8.6 (1.9-31.4) |
| Household substance use | 1.05 (0.94-1.17) | 1.01 (0.90-1.13) | NI |

Abbreviation: ACEs, adverse childhood experiences; AAEs, adverse adulthood experiences; HR, hazard ratio; 95% CI, 95% confidence interval; NI, not intermediate.

^a^ Models were adjusted for age, sex, residence, marital status, educational level, health behaviors (physical activity, smoking, and drinking), health status variables (BMI; history of diabetes, hypertension, dyslipidemia, and chronic kidney disease; use of diabetes medications, hypertension medications, and lipid-lowering therapy; and depressive symptoms), and adulthood social support.
