# Supplementary material for: Adverse childhood and adulthood experiences and risk of new-onset cardiovascular disease with consideration of social support: a prospective cohort study
Source: BMC Med. 2023 Aug 8;21:297. doi: 10.1186/s12916-023-03015-1 (PMC10408183; doi:10.1186/s12916-023-03015-1)
Supplement: Supplementary file 3 — Additional file 3: Figure S1. Overlapping exposure to adverse childhood experiences (ACEs) and adverse adulthood experiences (AAEs); Figure 2. Interaction and joint analysis of adverse childhood experiences (ACEs) and adverse adulthood experience (AAEs) with incident cardiovascular disease (CVD): subgroup analysis by sex; Figure S3. Interaction and joint analysis of adverse childhood experiences (ACEs) and adverse adulthood experience (AAEs) with incident cardiovascular disease (CVD): subgroup analyses: subgroup analysis by age; Figure S4. Interaction and joint analysis of adverse childhood experiences (ACEs) and adverse adulthood experience (AAEs) with incident CVD: using the complete dataset. [file 12916_2023_3015_MOESM3_ESM.docx]

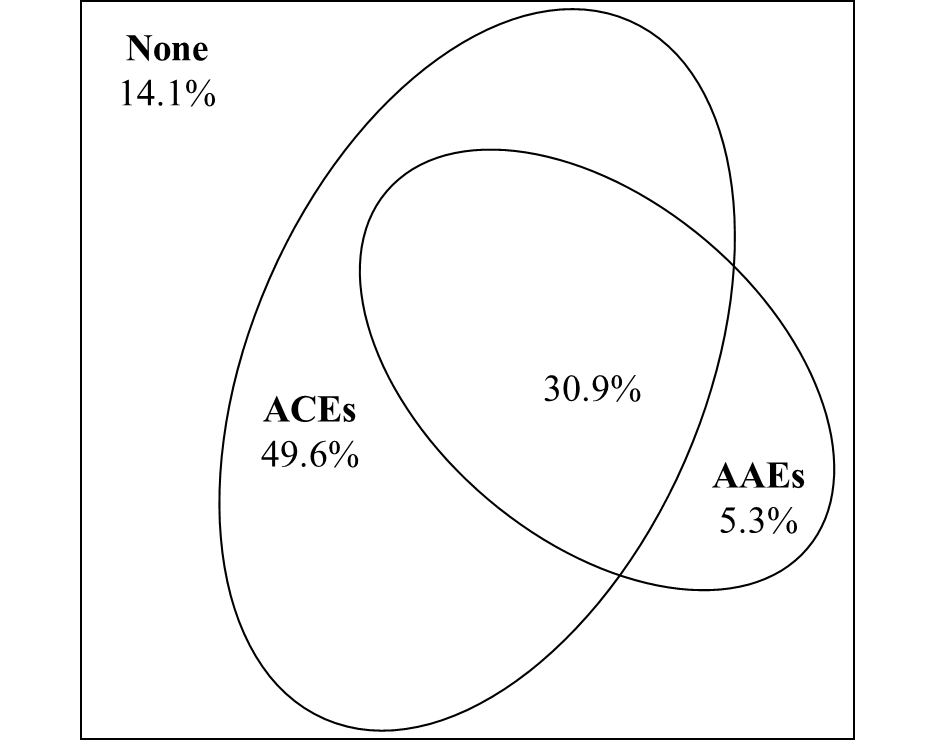


**Figure S1.** Overlapping exposure to adverse childhood experiences (ACEs) and adverse adulthood experiences (AAEs).


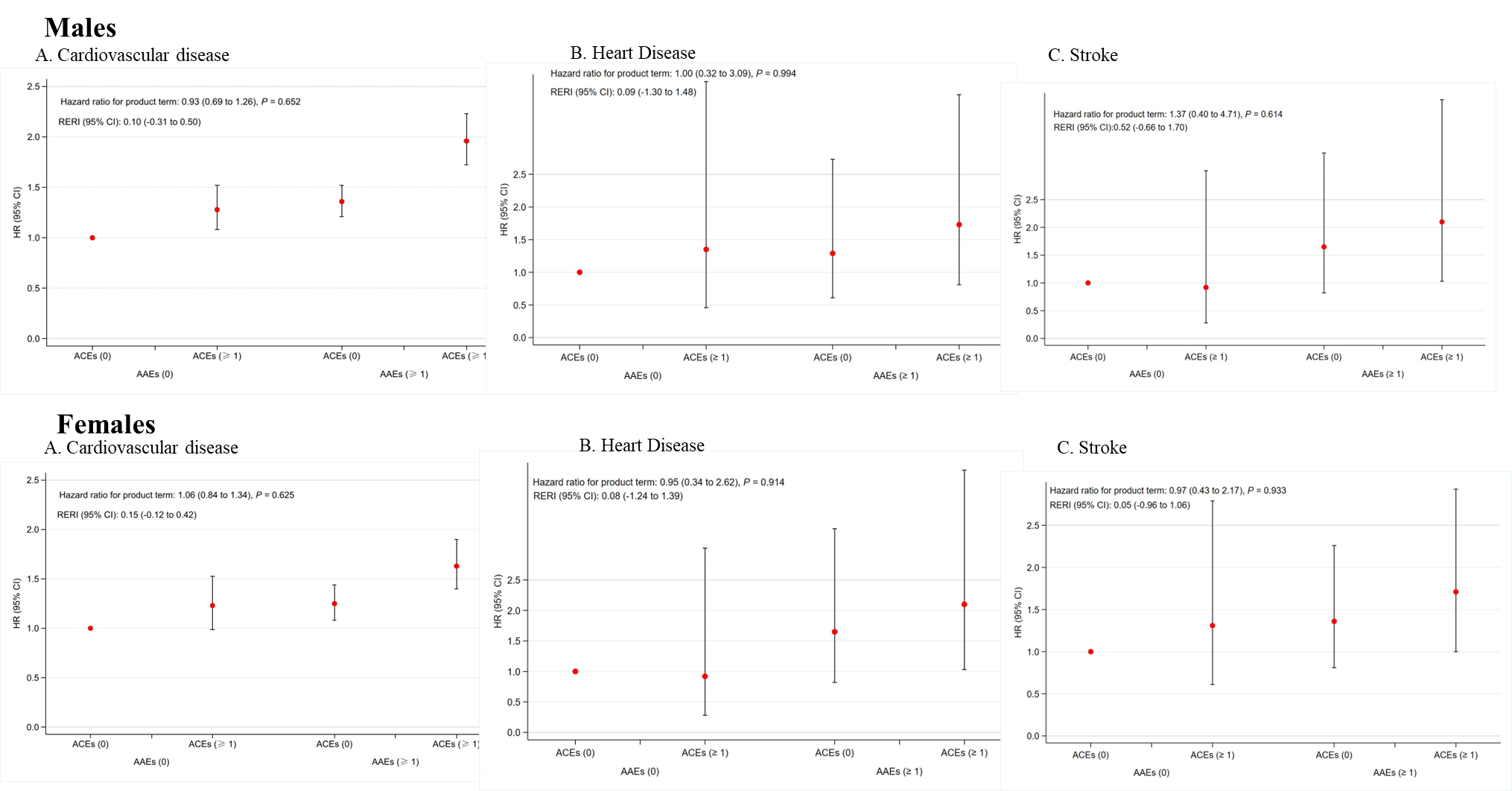


**Figure S2.** Interaction and joint analysis of adverse childhood experiences (ACEs) and adverse adulthood experience (AAEs) with incident cardiovascular disease (CVD): subgroup analyses by sex.


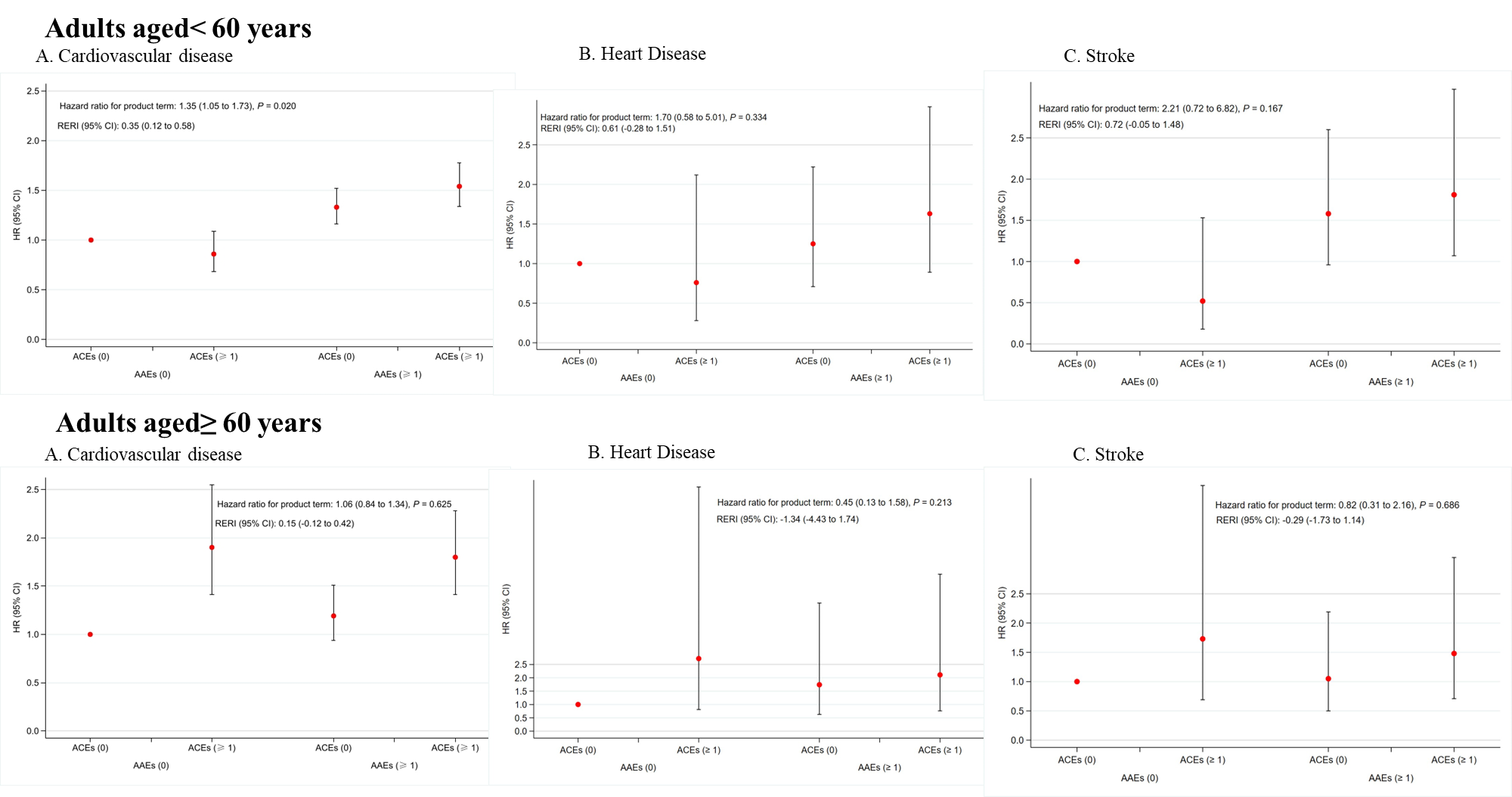


**Figure S3.** Interaction and joint analysis of adverse childhood experiences (ACEs) and adverse adulthood experience (AAEs) with incident cardiovascular disease (CVD): subgroup analyses by age.

**
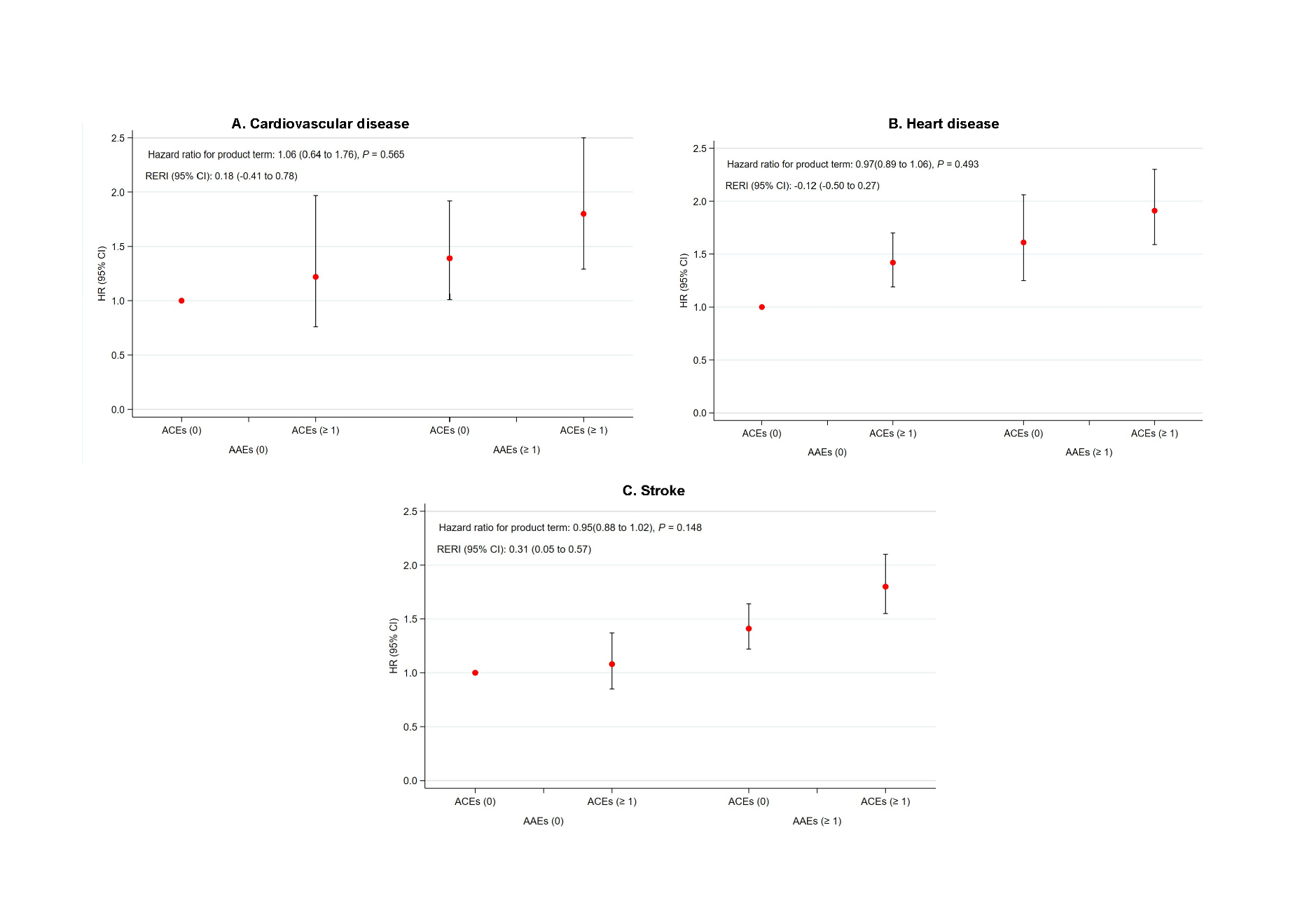
**

**Figure S4.** Interaction and joint analysis of adverse childhood experiences (ACEs) and adverse adulthood experience (AAEs) with incident CVD: using the complete dataset.
